# Overexpression of *OsPGIP2* confers *Sclerotinia sclerotiorum* resistance in *Brassica napus* through increased activation of defense mechanisms

**DOI:** 10.1093/jxb/ery138

**Published:** 2018-04-10

**Authors:** Zhuanrong Wang, Lili Wan, Qiang Xin, Ye Chen, Xiaohui Zhang, Faming Dong, Dengfeng Hong, Guangsheng Yang

**Affiliations:** 1 National Key Laboratory of Crop Genetic Improvement, Huazhong Agricultural University, Wuhan, Hubei, China; 2 Institute of Crop, Wuhan Academy of Agricultural Sciences, Wuhan, Hubei, China

**Keywords:** *Brassica napus*, defense pathways, *OsPGIP2*, damage/pathogen associated molecular patterns (DAMP/PAMP)-triggered immunity, polygalacturonase-inhibiting protein, polygalacturonase, resistance, *Sclerotinia sclerotiorum*

## Abstract

Sclerotinia stem rot (SSR), caused by *Sclerotinia sclerotiorum*, is the most serious disease affecting the yield of the agriculturally and economically important crop *Brassica napus* (rapeseed). In this study, *Oryza sativa* polygalacturonase-inhibiting protein 2 (*OsPGIP2*) was found to effectively enhanced rapeseed immunity against *S. sclerotiorum* infection. Leaf extracts of *B. napus* plants overexpressing *OsPGIP2* showed enhanced *S. sclerotiorum* resistance by delaying pathogen infection. The constitutive expression of *OsPGIP2* in rapeseed plants provided a rapid and effective defense response, which included the production of reactive oxygen species, interactions with *S. sclerotiorum* polygalacturonases (SsPG3 and SsPG6), and effects on the expression of defense genes. RNA sequencing analysis revealed that the pathogen induced many differentially expressed genes associated with pathogen recognition, redox homeostasis, mitogen-activated protein kinase signaling cascades, hormone signaling pathways, pathogen-/defense-related genes, and cell wall-related genes. The overexpression of *OsPGIP2* also led to constitutively increased cell wall cellulose and hemicellulose contents in stems without compromising seed quality. The results demonstrate that *OsPGIP2* plays a major role in rapeseed defense mechanisms, and we propose a model for *OsPGIP2*-conferred resistance to *S. sclerotiorum* in these plants.

## Teaser

Overexpression of rice *OsPGIP2* in *Brassica napus* results in resistance to *Sclerotinia sclerotiorum* by increasing activation of defense mechanisms and inhibiting fungal SsPG3 and SsPG6 proteins.

## Introduction

Sclerotinia stem rot (SSR) caused by the fungus *Sclerotinia sclerotiorum* (Lib.) de Bary, has a broad host range (Boland and Hall, 1994) and its severe symptoms make it the most devastating disease of rapeseed (*Brassica napus* L.). The fungus attacks rapeseed plants at all growth stages, but most especially in the flowering period, and it can infect leaves, stems, and siliques. Depending on the production area and climatology, SSR can cause severe yield losses ranging from 10–80% and results in low oil quality (Alizadeh *et al.*,2006; Sharma *et al.*, 2015). Despite the efforts of breeding programs, effective resistance sources against *S. sclerotiorum* have not yet been identified and the mechanisms underlying the interaction between rapeseed and *S. sclerotiorum* remain elusive. With the development of biotechnological tools, many candidate genes involved in *B. napus* defense against *S. sclerotiorum* have been identified (Zhao *et al.*, 2007; Wei *et al.*, 2016; Wu *et al.*, 2016). Transgenic manipulation of genes associated with innate defense signaling pathways may offer alternative approaches for improving resistance to *S. sclerotiorum* in rapeseed varieties.

During the interaction between plants and pathogens, two main immune responses provide natural disease-resistance mechanisms. Pathogen-associated molecular patterns (PAMP)-triggered immunity (PTI) is the primary defense response that activates downstream gene expression and results in relatively weak but race-non-specific hypersensitive responses (HRs) (Boller and Felix, 2009; Baxter *et al.*, 2014). Effector-triggered immunity (ETI) is induced by highly variable pathogen avirulence effectors that are recognized by host disease-resistance (R) proteins, and results in a rapid HR and long-lasting resistance (Boller and Felix, 2009). Both PTI and ETI can confer host-plant resistance to pathogens, but PTI overlapping with downstream immune responses is responsible for the responses against necrotrophs with a broad host range (Mengiste, 2012). The earliest defense reactions in PTI include the synthesis of reactive oxygen species (ROS), changes in ion fluxes across membranes, or increases in calcium (Ca^2+^) concentrations in the cellular space (Lecourieux *et al.*, 2006; Torres, 2010; Meng and Zhang, 2013; Baxter *et al.*, 2014; Seybold *et al.*, 2014). Calcium then acts as a diffusible second messenger for several resistance responses, including the synthesis of pathogen-related (PR) proteins and phytoalexins, and programmed cell death in neighboring cells (Zurbriggen *et al.*, 2009). Subsequent reactions include changes in plant endogenous hormone status, cell wall reinforcement by polysaccharide deposition and lignification, activation of downstream defense genes, and accumulation of secondary metabolites (Glawischnig, 2007; Peliter *et al.*, 2009; Matern *et al.*, 2011; Robert-Seilaniantz *et al.*, 2011; Chowdhury *et al.*, 2014).

During infection, pathogens secrete cell wall-degrading enzymes (CWDEs), such as pectinases, xylanases, polygalacturonases (PGs), and cellulases, in order to degrade cell wall polysaccharides and to penetrate and colonize host tissues (Bolton *et al.*, 2006; Fróes *et al.*, 2012). Concurrently, plants counteract these attacks with an array of inhibitor proteins (Juge, 2006). Interestingly, polygalacturonase inhibitor proteins (PGIPs) seem not only to recognize different surface motifs of functionally related structurally variable PGs but also to shift the breakdown process toward generating larger peptide fragments, which are damage-associated molecular pattern (DAMP)-active oligogalacturonides (OGs) (Federici *et al.*, 2006; Maulik *et al.*, 2012; Kalunke *et al.*, 2015). Transgenic *Arabidopsis thaliana* plants expressing a particular PGIP–PG chimera exhibit accumulated OGs and enhanced resistance to a variety of pathogens, providing direct evidence for the function of OGs as *in vivo* elicitors of plant defense responses (Benedetti *et al.*, 2015). Furthermore, treatment of plants with exogenous OGs promotes the production of ROS, up-regulates the expression of β-1,3-glucanase, increases the transcripts of phenylalanine ammonia-lyase (PAL) to accumulate phytoalexins and lignin (D’Ovidio *et al.*, 2004; Ridley *et al.*, 2001; Ferrari *et al.*, 2013), and promotes changes in gene expression in the salicylic acid (SA), ethylene (ET), and jasmonic acid (JA) pathways (Dupont *et al.*, 2006; Aziz *et al.*, 2007; Osorio *et al.*, 2008). Because the OG receptor WALL-ASSOCIATED KINASE 1 (WAK1) is capable of activating the kinase domain of the elongation factor Tu (EF-TU) receptor (EFR), OGs seem to be involved in PTI responses to necrotrophic pathogens (Mengiste, 2012).

The ‘PG–PGIP’ interaction mechanism provides a theoretical basis for plant disease resistance (Benedetti *et al.*, 2011; Gutierrez, 2012). Recent studies have demonstrated that overexpressing exogenous *PGIP*s is associated with a reduction of symptoms caused by bacteria, fungal pathogens, and insects in wheat, grape, apple, Chinese cabbage, tomato, and mung bean (Szankowski *et al.*, 2003; Agüero *et al.*, 2005; Hwang *et al.*, 2010; Schacht *et al.*, 2011; Borras-Hidalgo, *et al.*, 2012; Wang *et al.*, 2015; Chotechung *et al.*, 2016). In addition to *PvPGIP2* (*Phaseolus vulgaris*) and *BnPGIP2* (Akhgari *et al.*, 2012; Huangfu *et al.*, 2014), only a few other *PGIP* genes cause *S. sclerotiorum* resistance when overexpressed in *B. napus*. Although there are at least 16 *PGIP* genes that are highly induced by *S. sclerotiorum* infection in *B. napus* (Hegedus *et al.*, 2008), few commercial rapeseed varieties exhibit partial resistance (Wu *et al.*, 2016). *Arabidopsis thaliana* lines overexpressing *BnPGIP2* exhibit smaller necrotic lesions than wild-type plants, but no long-term effect on *S. sclerotiorum* disease progression is observed (Bashi *et al.*, 2013).

In *Oryza sativa*, the expression of *OsPGIP2* is induced more immediately and strongly at 0–12 h after *Rhizoctonia solani* infection than *OsFOR1*, which is the first *PGIP* gene identified with inhibitory activity against the PGase from *Aspergillus niger* (Jang *et al.*, 2003; Lu *et al.*, 2012). Therefore, Lu *et al.* (2012) proposed that increasing the expression of *OsPGIP2* by transgenic technology might correspondingly increase the resistance to pathogens in transgenic lines. In the present study, we transformed the exogenous *OsPGIP2* gene and introduced it into two different rapeseed backgrounds, and then assessed the enhancement of *S. sclerotiorum* resistance in the transgenic rapeseed lines. Our results indicated that the ectopic expression of *OsPGIP2* in rapeseed not only conferred resistance to *S. sclerotiorum* at both the seedling and adult stages, but also improved seed quality traits in the transgenic lines. Moreover, transient expression experiments showed that OsPGIP2 interacts with *S. sclerotiorum* SsPG3 and SsPG6 and, based on RNA sequencing analysis, we suggest that *OsPGIP2* played an important role in the defense mechanisms in the transgenic lines. Overall, our results demonstrate that *OsPGIP2* might be an effective gene for developing *S. sclerotiorum*-resistant rapeseed varieties.

## Materials and methods

### Plant transformation and growth conditions

The vector pCAMIBA1300-35S:*OsPGIP2* (kindly provided by Dr Liaoxun Lu, Huazhong Agriculture University, China) was transformed into the partially resistant *B. napus* line 7-5 and the susceptible line T45 by *Agrobacterium*-mediated transformation (Jonoubi *et al.*, 2005). Untransformed plants and transgenic lines were planted in an isolated nursery field at the Huazhong Agriculture University experimental farm in Wuhan, China.

### PCR and Southern blot analyses

Total genomic DNA was extracted from fresh leaves of wild-type and transformed plants using the cetyltrimethylammonium bromide (CTAB) method (Doyle, 1990). Verifications of *OsPGIP2* and the hygromycin resistance gene (*HYG*) in putative transgenic plants were performed by polymerase chain reaction (PCR) using the primers 333-L/R and 309-L/R. Each PCR was carried out in a 15-μl reaction that included 5 μM of each primer, 100 μM dNTP, 1× Taq buffer, 1.5 mM MgCl_2_, 2.0 units of Taq DNA polymerase (Thermo Scientific, Shanghai, China), and 50 ng of DNA template. PCR conditions consisted of 35 amplification cycles (94 °C for 30 s, 58 °C for 30 s, and 72 °C for 30 s) in total. PCR products were separated on a 1.0% (w/v) agarose gel and visualized after ethidium bromide staining. Southern blot analysis was carried out by the Towin biotechnology company (Wuhan, China) to confirm the integration of the introduced genes.

### Detached leaf inoculation

The PCR-positive transformed plants and untransformed controls were grown in the field to the 9–10 leaf stage. The eighth leaf of each individual plant was removed for detached leaf inoculation with *S. sclerotiorum* in a greenhouse, as previously described (Wu *et al.*, 2013). Ten biological replicates were performed for this experiment. At 48 h and 72 h post-inoculation (hpi), images were taken of inoculated leaves and lesion sizes were measured using ImageJ software (Nicholson, 2010).

### Hydrogen peroxide detection on inoculated leaves

The accumulation of H_2_O_2_ in leaves of transgenic plants was observed using 3′-3-diaminobenzidine (DAB) staining. Leaves inoculated with *S. sclerotiorum* were cut to the same size and stained with DAB solution as previously described (Dong *et al.*, 2008). Leaves were then washed in deionized water and treated with 96% ethanol to remove chlorophyll. Images were taken under a stereoscopic microscope (SZX16, Olympus, Japan).

### Test of seedling resistance under laboratory conditions

To prepare the *S. sclerotiorum* inoculum, five hyphal agar discs (8 mm in diameter) were placed into 100 ml of PDB (potato-dextrose broth) liquid medium for 3 d at 22 ± 2 °C under continuous shaking (200 rpm). Hyphae were then disrupted in a blender to produce a 5% (v/v) *S. sclerotiorum* hyphal suspension, which was sprayed onto 6-week-old seedlings at 100 ml m^–2^. For each rapeseed line, approximately 20 seedlings were planted in 1-l pots, using three biological replicates. Lines 7-5WT and T45WT were used as untransformed controls; ZS11 (Zhongshuang11) and Westar were used as resistant and susceptible controls, respectively. Inoculated plants were incubated in a growth chamber at 22 ± 2 °C under 16 h light/8 h dark and 80% relative humidity. At 14 d post-inoculation (dpi), we counted the number of seedlings that were completely withered (dead), that were diseased but not wilting (susceptible), and that were without symptoms (healthy).

### Design of field trial and stem inoculation at the flowering stage in the field

Six randomized-block replicates, consisting of 12 rows of approximately 120 plants per transgenic line, were used in field experiments. Two blocks were selected for stem inoculation of *S. scleroterum*, two were selected to measure artificial infection by the fungus, and the remaining two were not subject to any pathogen inoculation. Lines 7-5WT and T45WT were used as untransformed controls. Crop management followed the standard agronomic practice excluding pesticide application. Field stem inoculations were carried out using a method modified from Wu *et al.* (2013). Agar discs (8 mm in diameter) were excised from the edges of growing fungal colonies and up-ended into the lids of 1.5-ml or 2.0-ml centrifuge tubes, which were then affixed with plastic wrap onto rapeseed stems 50 cm from the ground. Disease severity was assessed by measuring lesion length per pathogen infection spot at 7 dpi. Inoculation, observation, and disease resistance were only determined for plants located in the center of blocks to avoid marginal effects.

### Artificial infection using *S. sclerotiorum* suspension and evaluation of disease resistance

In the two randomized blocks selected to measure artificial infection, a 5% suspension of *S. sclerotiorum* hyphae was sprayed onto rapeseed plants at the full-flowering stage. Lines 7-5WT and T45WT were used as untransformed controls; ZS11 and Westar were used as resistant and susceptible varieties, respectively. At 7 d before harvest, disease severity was assessed and classified as follows: 0, no lesions; 1, superficial lesions or small branches affected; 2, large branches dead; 3, at least 50% of main stem circumference with lesions; 4, main stem girdled but plant produced good seeds; 5, main stem girdled and much reduced yield, or dead (Falak *et al.*, 2011). The disease index (DI) was calculated as 100[∑(*N*_*i*_*i*)]/(*kN*), where *i* is the disease severity score, *k* is the highest score that was observed, *N*_*i*_ is the number of every line with each score, and *N* is total number of plants assessed per line (Liu *et al.*, 2005). The relative resistance index (RRI) was calculated as ln[(100−*DI*_ck_)/*DI*_ck_]−ln[(100−*DI*_m_)/*DI*_m_] where *DI*_ck_ is the disease index of the control (variety ZS11), and *DI*_m_ is that of the line being evaluated. The relative resistance was then evaluated as: highly resistant (HR), RRI≤–1.2; moderately resistant (MR), –1.2<RRI≤–0.7; lightly resistant (LR), –0.7<RRI≤0; lightly susceptible (LS), 0<RRI≤0.9; moderately susceptible (MS), 0.9<RRI≤2.0; and highly susceptible (HS), RRI>2.0 (Liu *et al.*, 2005).

### Analysis of seed quality traits

We harvested seed from fully open-pollinated rapeseed plants in the inoculated blocks. Nine plants were harvested from every line in the center of each plot. The 1000-seed weight from each line was calculated by averaging three measurements of 1000 well-filled seeds dried naturally for at least 3 weeks after harvest (Fan *et al.*, 2010). Near-infrared reflectance spectroscopy (NIRS) (FOSS Analytical A/S, NIRS, USA) was used to measure the contents of protein, glucosinolate (GSL), oil, and erucic acid (Kumar *et al.*, 2010).

### Antifungal activity assay *in vitro*

Fresh leaves (5 g) were ground to powder in liquid nitrogen and then mixed with 30 ml extraction buffer (50 mM MES, 100 mM TrisHCl, 0.1 mM EDTA, 30 mM NaCl, pH=7.0) (Jiang *et al.*, 2013). This mixture was then centrifuged at 10 000 *g* for 10 min at room temperature and the supernatant was collected as the crude leaf extract. A *S. sclerotiorum* mycelia plug was inoculated onto the center of a potato dextrose agar (PDA) medium, which was mixed with 50% (v/v) crude leaf extracts from transgenic lines or extraction buffer (control) in three biological replicates. These mixtures were incubated in the dark for 36 h before hyphae were visualized under a stereoscopic microscope (SZX16, Olympus, Japan).

### Determination of cell wall monosaccharides in stems

The stems of rapeseed plants at the flowering and final flowering stages were cut 10–50 cm above ground and the leaves were removed. Three biological replicates were used in this assay. The stem tissue was completely dried at 60 °C, crushed in a pulverizer, and passed twice through a 60-mesh sieve (0.3 mm). Samples were first extracted with benzene-ethanol Soxhlet extractor to remove resin and pigments, and then hydrolyzed with 72% H_2_SO_4_ and 4% H_2_SO_4_ to obtain the lignocellulose components that are easy to quantify, including cellulose and hemicellulose. These were hydrolyzed to monosaccharides, which were quantified by high-performance liquid chromatography (HPLC, LC-20AT, Shimadzu, Japan); the content of acid-insoluble lignin was determined by the burning method and acid-soluble lignin was determined by UV-Visible spectrophotometry (UV-2550) (Sluiter *et al.*, 2004).

### RNA extraction and RNA- sequencing analysis of differentially expressed genes

At five time-points (0, 1, 3, 5, and 7 dpi), epidermal stem tissues extending 5 mm beyond the inoculation site and 1 mm deep were excised using a razor blade. Tissues were harvested from nine plants in the two stem-inoculated blocks in the field, and three harvested plants were pooled to form each sample, generating three biological replicates for quantitative real-time PCR (qPCR). Total RNA was extracted using an RNeasy Plant Mini Kit, (Promega, shanghai, China), according to the manufacturer’s instructions. For RNA sequencing (RNA-seq), equal RNA concentrations from three biological replicates at 3 dpi were pooled into a single sample, and samples of 7-5WT, 7-5D, T45WT, and T45B#2 were sequenced on an Illumina Hiseq 2000 platform (Novogene Bioinformatics Technology Co., Ltd, Beijing, China). The mRNA sequencing produced 39 654 044 (T45WT), 43 614 190 (T45B#2), 54 035 950 (7-5WT), and 47 130 844 (7-5D) clean raw reads. To identify genes corresponding to reads from each sample library, the reads were aligned to the *B. napus* reference genome (Chalhoub *et al.*, 2014) and the *S. sclerotiorum* genome (Amselem *et al.*, 2011) using TopHat (Trapnell *et al.*, 2012). Gene expression levels were estimated based on fragments per kilobase of exon per million fragments mapped (FPKM), which were used to draw heatmaps for the differentially expressed genes (DEGs) identified with DEGseq based on the criteria *q*-value <0.005 and |log2 (FPKM-transgenic/FPKM-WT)| >1 between transgenic and wild-type lines (Wang *et al.*, 2010). For gene ontology (GO) term annotations, all *B. napus* genes were searched against the National Center for Biotechnology Information (NCBI) non-redundant (Nr) protein database using GOSeq with corrected *P*-value <0.05 (Young *et al.*, 2010). The transcriptome data has been deposited in NCBI Sequence Read Archive database (accession number, SRP145538).

### Quantitative real-time polymerase chain reaction analysis

qPCR was performed on an ABI 7500 Real-Time PCR System using GO Taq® qPCR Master Mix (Promega) according to the manufacturer’s instructions. The primers used for amplifications are listed in Supplementary Table S4 at *JXB* online. Cycle threshold (*C*_t_) values and relative abundance were calculated using the relative quantification method (Livak and Schmittgen, 2001). The genes *BnACTIN7* (*AF111812*) and *UBC21* (ubiquitin-conjugating enzyme 21) were used as references for the analysis of rapeseed gene expression. 28S ribosomal DNA (*AF431951.1*) and *H3* (histone 3) were used as internal controls for *S. sclerotiorum* gene expression. Three biological replicates and three technical replicates were performed for each sample.

### expression assay

The affinities between OsPGIP2–SsPGs were predicated using PPA-Pred2 (Protein–Protein Affinity Predictor; https://www.iitm.ac.in/bioinfo/PPA_Pred/prediction.html). The class of the protein–protein complex was set as ‘Enzyme-Inhibitor’. The smaller the dissociation constant, the more tightly bound the ligand is, or the higher the affinity between ligand and protein. Transient expression assays were performed in leaves of tobacco (*Nicotiana benthamiana*) as previously described (Li *et al.*, 2013). The coding sequences of *SsPG1* (*AF501307*), *SsPG3* (*AY312510*), *SsPG5* (*AY496277*), and *SsPG6* (*AF501308*) were subcloned into the N-terminal luciferase-fusion vector JW771-NLUC. The coding sequence of *OsPGIP2* (*AM180653.1*) was sub-cloned into the C-terminal luciferase-fusion vector JW772-CLUC. Relevant primer sequences are listed in Supplementary Table S4. For protein interaction analysis, the plasmids of two combinatory constructs were mobilized into *Agrobacterium tumefaciens* and transformed simultaneously into the leaves of 5-week-old tobacco plants using a needleless syringe (Sparkes *et al.*, 2006). After infiltration, plants were incubated at 22 °C for 48 h before the leaf surface was sprayed with 100 mM Beetle luciferin potassium salt (Promega, Madison, WI, USA) and kept in darkness for 5 min. Luminescent images were captured using a Lumazone 1300B imaging apparatus with a cool charge-coupled device CCD (iXon; Andor Technology, Belfast, UK). Two biological replicates with two technical replicates were used in the assays.

### Computational and statistical analyses

Quality traits of seeds, 1000-seed weight, cell wall monosaccharides of stems, lesion length, lesion area from the different transgenic and WT rapeseed lines, and correlations between qPCR and RNA-seq were analysed using SPSS version 22.0. Where the statistical test for significance was between two groups, one-way ANOVA was used, but when comparing between three or more groups, least-significant difference (LSD) multiple-comparison tests were used.

## Results

### Stable transgenic *B. napus* seedlings overexpressing *OsPGIP2* are resistant to *S. sclerotiorum*

**Fig. 1.**
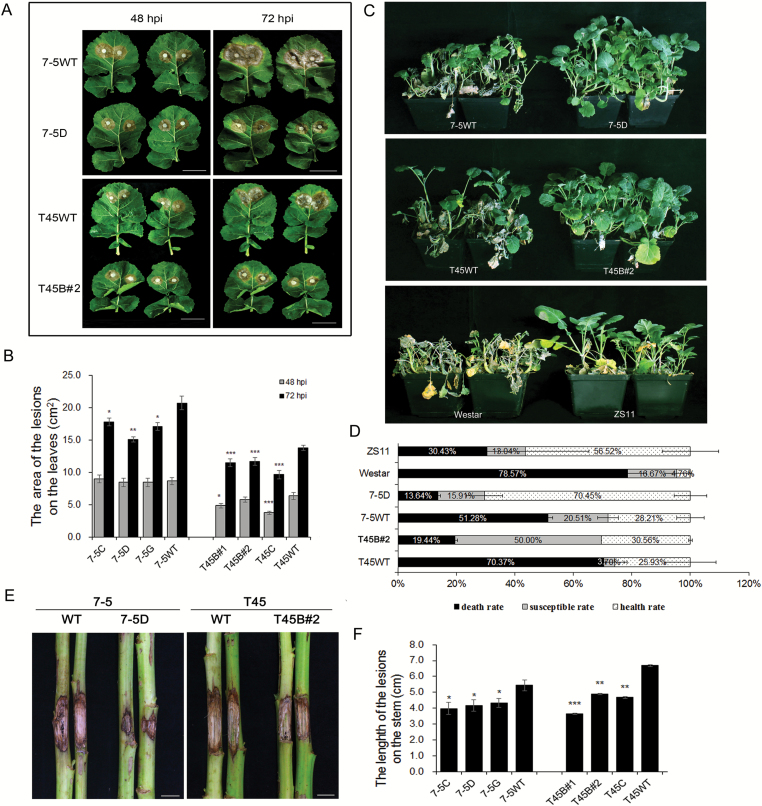
*OsPGIP2* overexpression conferred resistance to *S. sclerotiorum* in transgenic rapeseed plants. (A) T_4_ homozygous lines (7-5D and T45B#2) and wild-types (WT) inoculated with 8-mm agar plugs of *S. sclerotiorum* hyphae at 22 °C. Images were taken at 48 and 72 h post-inoculation (hpi). Scale bars are 10 cm. (B) Lesion areas in transgenic and untransformed rapeseed plants at 48 and 72 hpi. Data mean values (±SE) (*n*=20), and significant differences were determined by one-way ANOVA: ***P<0.001, **P<0.01, *P<0.05. 7-5WT and T45WT are wild-type controls. (C) Seedlings of 6-week-old transgenic and control *B. napus* plants sprayed with hyphal solution. Infection phenotypes were assessed at 14 d post-inoculation. ZS11 was the resistant control and Westar was the susceptible control. 7-5WT and T45WT are wild-type controls. (D) Analysis of the survival of transgenic lines inoculated with *S. sclerotiorum* hyphae. ZS11 was the resistant control and Westar was the susceptible control. Data are means of three biological replicates (±SE). 7-5WT and T45WT are wild-type controls. (E) Stems of transgenic lines and wild-types 1 week after inoculation with *S. sclerotiorum*. Scale bars are 2 cm. (F) Stem lesion lengths in transgenic *OsPGIP2*-overexpressing lines and wild-types 1 week after inoculation with *S. sclerotiorum*. 7-5WT and T45WT are wild-type controls. Data are means (±SE) (*n*≥20). Significant differences were determined by one-way ANOVA: ***P<0.001, **P<0.01, *P<0.05.

We constitutively expressed *OsPGIP2* in the partially resistant *B. napus* line 7-5 and the susceptible line T45, and the transgenic plants did not show any major morphological or growth defects (see Supplementary Fig. S2A, B). The T_4_ homozygous *OsPGIP2*-expressing transgenic lines 7-5C, 7-5D, 7-5G, T45B#1, T45B#2, and T45C, which contained a single copy of *OsPGIP2* as confirmed by Southern blotting (Supplementary Fig. S1A), were selected for further analyses. qPCR analysis of these lines revealed that the *OsPGIP2* gene was expressed in all independent transgenic lines (Supplementary Fig. S1B); the PCR analysis showed that *OsPGIP2* and *HYG* were stably inherited in these transgenic lines up to the T_4_ generation (Fig. S1C, D). To test the resistance to *S. sclerotiorum*, we used leaves detached from T_4_ seedlings at 48 and 72 hpi (Fig. 1A). In the 7-5 background, no significant differences in the area of necrotic lesions were detected between the transgenic lines and the wild-type at 48 hpi; however, at 72 hpi, the 7-5C and 7-5G lines had smaller necrotic lesions than the untransformed plants (*P*<0.05), and the 7-5D lines were significantly resistant to infection compared to inoculated untransformed leaves (*P*<0.01; Fig. 1B). In the T45 background, only T45B#2 was not significantly different from its control after 48 hpi; after 72 hpi, all the transgenic lines showed a significant reduction in lesion size compared to the inoculated wild-type (*P*<0.001; Fig. 1B). Moreover, after spraying the leaves of 6-week-old transgenic lines (7-5D and T45B#2) with hyphae suspended in PDB liquid medium, those from wild-type plants dropped and died while most of the lines overexpressing *OsPGIP2* exhibited normal growth phenotypes (Fig. 1C). The transgenic line 7-5D showed a decreased death rate (13.64%) compared to 7-5WT (51.28%); T45B#2 lines had a 19.44% death rate while T45WT presented a death rate of 70.37% (Fig. 1D). Thus, between 48 and 72 hpi, the necrotic lesions in the transgenic lines expanded more slowly than in control plants, and the transgenic lines showed a higher survival rate than the wild-type lines when sprayed with hyphae. These results suggest that *OsPGIP2* enhanced rapeseed resistance to *S. sclerotiorum* at the seedling stage under controlled disease stress conditions.

### Transgenic rapeseed lines respond to *S. sclerotiorum* infection in the field

At the flowering stage, the primary stems of transgenic rapeseed lines were inoculated with isolated *S. sclerotiorum* using hyphal agar plugs. After 7 d, all transgenic individuals in the 7-5 and T45 backgrounds showed smaller lesions on their stems compared with wild-type plants (Fig. 1E, F). Resistance under field conditions was assessed in three consecutive self-crossed generations (2013–2016) and remained relatively consistent throughout the years (see Supplementary Table S1). After plants were sprayed with *S. sclerotiorum* hyphae in the field, RRI screening revealed that transgenic lines 7-5D and T45B#1 were moderately resistant (MR) compared to their control lines; 7-5WT was moderately susceptible (MS), and T45WT was highly susceptible (HS) compared to the ZS11 resistant control (Table 1).

**Table 1.** Resistance assessment of T_4_ homozygous transgenic rapeseed lines under controlled disease pressure in the field

Variety	DI^a^	RRI^b^	Control	Category^b^
ZS11	0.09	0.00	ZS11	–
Westar	0.89	2.31	ZS11	HS
7-5C	0.43	–0.42	7-5WT	LR
7-5D	0.31	–0.73	7-5WT	MR
7-5G	0.52	–0.23	7-5WT	LR
7-5WT	0.65	1.99	ZS11	MS
T45B#1	0.42	–0.74	T45WT	MR
T45B#2	0.74	–0.17	T45WT	LR
T45C	0.52	–0.52	T45WT	LR
T45WT	0.87	2.29	ZS11	HS

Data were collected at the harvest stage, after spraying plants (*n*>30) with *S. sclerotiorum.* WT indicates non-transformed rapeseed plants. ZS11 was the resistant cultivar control and Westar was the susceptible cultivar control.

^a^ DI is the *S. sclerotiorum* disease incidence measured under controlled disease stress in field conditions, and is classified from 0 (low) to 5 (high).

^b^ RRI is the relative disease resistance, and is the categories are classified as high (HR), medium (MR), or low (LR) resistance, or as low (LS), medium (MS), or high (HS) susceptibility.

Because the known detrimental effects of SSR on rapeseed, we assessed seed quality traits and determined the grain weight of transgenic and wild-type plants following stem infection and disease development. Although the T_4_ homozygous transgenic *OsPGIP2* lines did not show any significant differences in seed protein or erucic acid contents compared with the wild-type plants after inoculation, the GSL content displayed a significant decrease in the transgenic lines (Table 2). In the 7-5 genetic background, the 1000-seed weight of the transgenic lines ranged from 2.99–3.25 g, which was higher than that of wild-type plants (1.62 g). The oil content was 38.38–40.05% higher than that of the wild-type lines (30.68%) (Table 2). In individual T45 transgenic plants, seed weight ranged from 2.75–3.29 g, which was greater than that of the wild-type plants (1.68 g). The oil content of the T45 transgenic lines was 38.72–40.42% while that of the untransformed line was 30.26% (Table 2). In summary, these measurements were consistent with the results of the previous *S. Sclerotiorum* hyphal treatment (Table 2, and see Supplementary Fig. S3), and indicated that transforming *OsPGIP2* into rapeseed could maintain seed quality traits in plants exposed to *S. sclerotiorum*. In general, all the transgenic lines showed stable seed quality and improved resistance to *S. Sclerotiorum* infection in the adult stage under field conditions.

**Table 2.** Quality traits of seeds of transgenic rapeseed lines evaluated after S. sclerotiorum inoculation in the field in 2016

Variety	1000-seed weight (g)	Oil (%)	Glucosinolate (µmol g^–1^)	Protein (%)	Erucic acid (%)
7-5C	2.99 ± 0.06^a^	40.05 ± 1.87^a^	15.31 ± 0.25^c^	26.69 ± 0.28^a^	0.77 ± 0.17^a^
7-5D	3.25 ± 0.15^a^	39.74 ± 0.25^a^	17.39 ± 0.83^b^	26.45 ± 0.35^a^	0.79 ± 0.06^a^
7-5G	3.07 ± 0.13^a^	38.38 ± 0.24^a^	13.85 ± 0.73^d^	27.89 ± 0.15^a^	0.75 ± 0.06^a^
7-5WT	1.62 ± 0.08^b^	30.68 ± 0.45^b^	22.46 ± 1.61^a^	28.52 ± 0.26^a^	0.70 ± 0.07^a^
T45B#1	2.75 ± 0.06^b^	38.99 ± 0.47^a^	16.16 ± 1.24^d^	26.67 ± 0.26^a^	0.75 ± 0.04^a^
T45B#2	3.29 ± 0.24^a^	38.72 ± 0.69^a^	23.61 ± 3.08^b^	27.87 ± 0.39^a^	0.91 ± 0.08^a^
T45C	2.97 ± 0.04^a^	40.42 ± 0.19^a^	21.03 ± 1.11^c^	26.34 ± 0.22^a^	0.77 ± 0.08^a^
T45WT	1.68 ± 0.11^c^	30.26 ± 0.06^b^	34.45 ± 0.12^a^	27.41 ± 0.04^a^	0.84 ± 0.04^a^

The field assay in the T_4_ generation was performed in two completely randomized blocks and each replicate contained about 20 plants. WT indicates non-transformed rapeseed plants. Values are means of nine replicates ±SD. Seed quality traits were measured based on 1000 well-filled dry seeds. Multiple comparisons were used to test statistical significance. Different letters indicate significant differences at *P*<0.05.

### Leaf extracts of *OsPGIP2* transgenic lines delay *S. sclerotiorum* growth

**Fig. 2.**
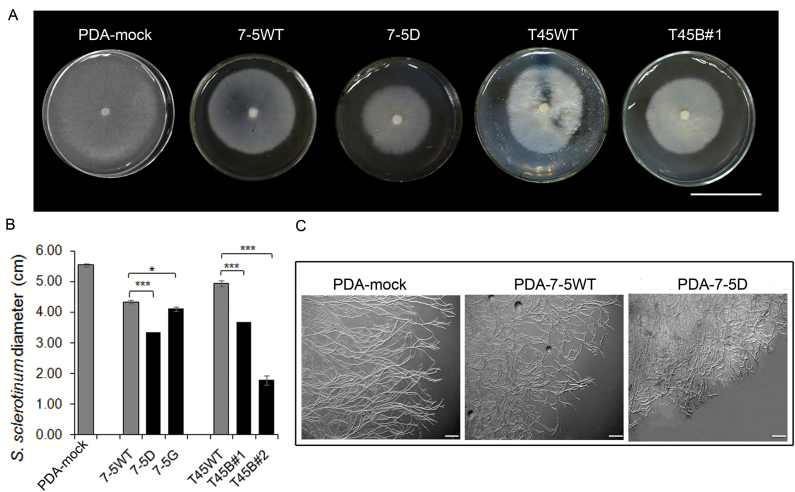
Cultures of *S. sclerotiorum* on PDA plates with leaf extracts from *OsPGIP2* transgenic and wild-type lines. (A) Images of *S. sclerotiorum* inoculated on PDA plates treated with either leaf extracts (50%) or a mock solution at 36 h post-infection (hpi). Scale bars are 4 cm. (B) Statistical analysis of the diffusion diameter of *S. sclerotiorum* on PDA plates treated with either leaf extracts (50%) or a mock solution at 36 hpi. Data are means from three biological replicates (±SD) (*n*=3). Significant differences were determined by one-way ANOVA: ***P<0.001, **P<0.01, *P<0.05. 7-5WT and T45WT are wild-type controls. (C) Hyphal growth of *S. sclerotiorum* on PDA plates treated with either *OsPGIP2* transgenic leaf extracts (50%) or a mock solution at 36 hpi. Scale bars are 1 mm.

To verify resistance *in vitro*, we cultivated *S. sclerotiorum* in uninfected transgenic *OsPGIP2* rapeseed leaf extracts (Fig. 2A). We found that *S. sclerotiorum* grew significantly more on PDA without leaf extract than when cultured with 50% (v/v) leaf extract from transgenic lines and wild-type plants; however, the diameters of the *S. sclerotiorum* colonies in PDA with leaf extracts from transgenic rapeseed were significantly smaller than in PDA with control leaf extract (Fig. 2B). In addition, whilst the hyphal growth of *S. sclerotiorum* was evenly and sparsely distributed on the PDA plates with extraction buffer (control) (Fig. 2C), in the presence of leaf extracts from transgenic and wild-type lines growth was reduced, with dense distribution and entangled ends of the hyphae. The reduction in growth was more conspicuous on PDA plates containing leaf extracts from the transgenic lines (Fig. 2C). These experiments suggested that leaf extracts of transgenic lines might effectively delay the expansion of *S. sclerotiorum*.

### DEGs in transgenic plants infected with *S. sclerotiorum*

**Fig. 3.**
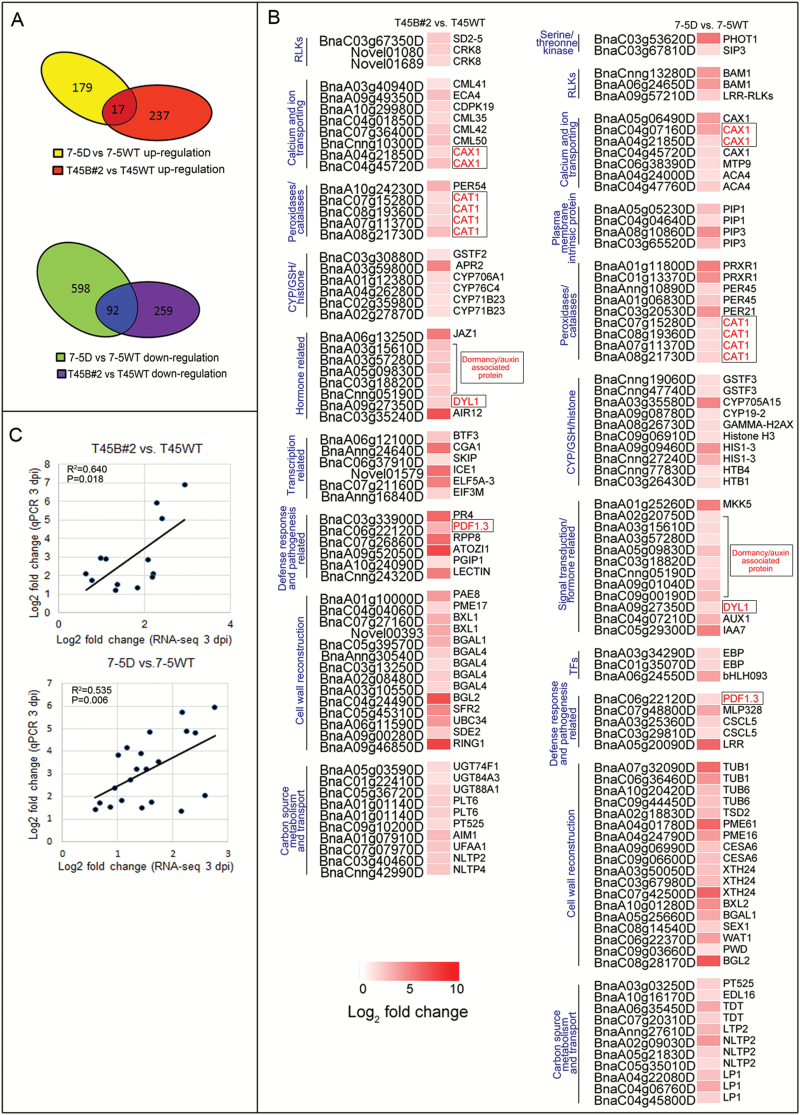
Differential gene expression between transgenic and wild-type *B. napus* lines in response to *S. sclerotiorum* infection. (A) Venn diagrams representing the up- and down-regulation of *B. napus* differentially expressed genes (DEGs) in response to *S. sclerotiorum* infection at 3 d post-inoculation (dpi). (B) Heatmap of a subset of significantly up-regulated DEGs related to *S. sclerotiorum* infection. DEGs were considered statistically significant if *q*-value <0.005 and |log_2_-fold change| >1. The log_2_-fold change is indicated according to the scale bar. The intensity of the colour indicates the expression of genes, with a darker colour meaning higher gene expression. The common up-regulated DEGs are highlighted by boxes. Details of the genes are given in Supplementary File S2. (C) Correlations of gene expression ratios obtained by RNA-seq and qPCR. The qPCR log_2_-value of the expression ratio (transgenic lines-inoculated/WT-inoculated) (*y*-axis) was plotted against the value from the RNA-seq (*x*-axis). All qPCR data were collected from three biological replicates and three technical replicates for each sample, and RNA-seq data were obtained from one pooled sample.

To elucidate the molecular network underlying *OsPGIP2* disease resistance, we performed transcriptome profiling analysis on the stems of transgenic (7-5D and T45B#2) and wild-type (7-5WT and T45WT) lines at 3 dpi (Supplementary File S1). The proportions of reads mapped to the *S. sclerotiorum* genome were significantly higher in the wild-type than in the transgenic lines (see Supplementary Table S2), suggesting that *S. sclerotiorum* could infect and propagate more easily in the wild-type lines. To explore the biological functions of *OsPGIP2* in 7-5D and T45B#2, reads were mapped to the reference genome of *B. napus* (Chalhoub *et al.*, 2014). We found that 17 up-regulated genes were common to both genotypes, 179 genes were specific to the 7-5 background, and 237 were specific to the T45 background (Fig. 3A). There were 92 down-regulated genes that were common to both genotypes; 598 were specific to 7-5, and 259 were specific to T45. To validate the data obtained from the RNA-seq, 13 and 21 genes from the T45 and 7-5 transgenic lines, respectively, were selected for qPCR. For all 34 genes, the results obtained using both techniques were highly related at 3 dpi (T45B#2: *R*^2^=0.640, *P*<0.018; 7-5D: *R*^2^=0.535, *P*=0.006), confirming the reliability of RNA-seq data (Fig. 3C). In addition, 18 genes were also tested at 0–7 dpi in the T45 and 7-5 backgrounds, and this revealed their higher expression levels in transgenic than in non-transgenic lines after inoculation (Supplementary Figs S6, S7).

### GO analysis reveals the defense mechanisms of *OsPGIP2* transgenic lines to *S. sclerotiorum*

Gene ontology (GO) term annotations were performed to identify any significant enrichment in each term relative to the entire proteome (see Supplementary Fig. S4B, C). Although T45B#2 and 7-5D showed a similar pathway to enhanced SSR resistance, comprising 17 up- and 92 down-regulated shared genes, genes in the same category were largely different (Fig. 3B, Supplementary Fig. S5). In 7-5D compared with 7-5WT, we identified three up- and six down-regulated receptor-like protein kinases (RLKs), which participate in intracellular protein interactions and signal transduction, and function in PAMP recognition (Zhang *et al.*, 2013) (Supplementary Fig. S5, Fig. 3B). However, comparing transgenic and wild-type in the T45 background, we identified three up- and three down-regulated RLKs, and these DEGs are different from the RLKs in the 7-5 background (Supplementary Fig. S5, Fig. 3B). Eight hormone-related genes were up-regulated in T45B#2 compared to the T45WT line, including *JAZ1* (jasmonate-zim-domain protein 1) and seven auxin-associated genes (Fig. 3B). For signal transduction, three mitogen-activated protein (MAP) kinases were down-regulated and MAP kinases (MKK5) were up-regulated in the 7-5D transgenic line compared to 7-5WT (Supplementary Fig. S5, Fig. 3B). After *S. sclerotiorum* infection, defense-related genes and transcription factors (TFs) also were induced in the *OsPGIP2*-expressing lines; for example, plant defensin 1.3 (*PDF1.3*), pathogenesis-related 5 (*PR-5*), and *WRKYs* were induced in the 7-5D and T45B#2 transgenic lines (Fig. 3B, Supplementary S5). Within the ‘cell wall reconstruction’ pathway, the most prominent changes in expression included the up-regulation of xyloglucan endotransglucosylase/hydrolase protein 24 (*XTH24*) and pectinesterase family proteins *PME61*, and the down-regulation of arabinogalactan (*AGP20*) in 7-5D (Fig. 3B, Supplementary S5). These cell wall-modifying enzymes modulate cell wall extensibility and plasticity during both developmental and stress-response programs (Le Gall *et al.*, 2015). Based on the distribution of all the DEGs, up- and down-regulated genes in the transgenic lines responding to *S. sclerotiorum* were classified into plant defense-response pathways, including serine/threonine kinase and RLKs, calcium and ion transporting, peroxidases/catalases, signal transduction/hormone-related, pathogen-related defense response, transcription-related, cell wall reconstruction, cytochrome/glutathione/histone (CYP/GSH/histone), and carbon source metabolism and transport (Fig. 3B, Supplementary S5).

### *S. sclerotiorum* infection induces H_2_O_2_ accumulation in *OsPGIP2* transgenic lines

**Fig. 4.**
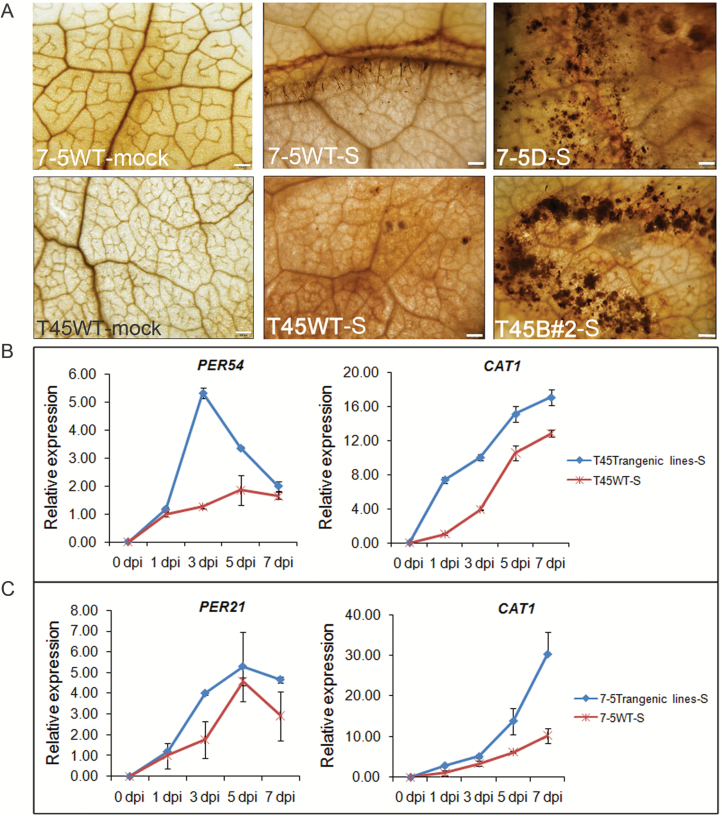
H_2_O_2_ accumulation and the expression levels of H_2_O_2_-associated genes in response to *S. sclerotiorum* inoculation in *OsPGIP2*-overexpressing and wild-type lines of rapeseed. (A) Detection of H_2_O_2_ accumulation by DAB staining in *B. napus* leaves after *S. sclerotiorum* inoculation. The images show leaves at 72 h post-infection with either mock solution or *S. sclerotiorum* (S). Seedlings with 9–10 leaves were used and the inoculation assays were conducted on detached leaves. Three biological replicates were used in this experiment. Scale bars are 500 μm. (B, C) Expression levels of *PER21* (*BnaC03g20530D*), *PER54* (*BnaA10g24230D*), and *CAT1* (*BnaA07g11370D*) in wild-type (WT) and *OsPGIP2*-expressing transgenic lines inoculated with *S. sclerotiorum* as determined by qPCR and normalized to *BnACTIN7* and *UBC21*. Data are means (±SD) and are representative of three biological and three technical replicates. dpi, days post-inoculation.

As a second messenger, ROS directly and indirectly regulates the innate immune system of plants to enhance defense against disease (Foyer and Noctor, 2011). In our experiments, more peroxidase and catalase genes were differently expressing in transgenic and control plants after *S. sclerotiorum* inoculation (Fig. 3B, Supplementary S5). The difference in ROS (H_2_O_2_-derived) production between transgenic and wild-type plants was detected by DAB staining. Inoculated sites on transgenic 7-5D and T45B#2 leaves infiltrated with *S. sclerotiorum* were more strongly stained by DAB than controls (Fig. 4A), indicating that H_2_O_2_ accumulation in *OsPGIP2*-expressing leaves was strongly induced by infection. After *S. sclerotiorum* inoculation, the expression of ROS-related enzymes such as *PER21* (peroxidase 21, *BnaC03g20530D*), *PER54* (*BnaA10g24230D*), and *CAT1* (catalase 1, *BnaA07g11370D*) increased significantly in both the transgenic and wild-type plants, but this increase was more significant in the *OsPGIP2* transgenic plants than in the wild-type controls (Fig. 4B, C). These data indicated that *OsPGIP2* transgenic plants accumulated more H_2_O_2_ to increase innate immune signaling and to enhance plant resistance to *S. sclerotiorum*.

### OsPGIP2 directly interacts with SsPG3 and SsPG6

**Fig. 5.**
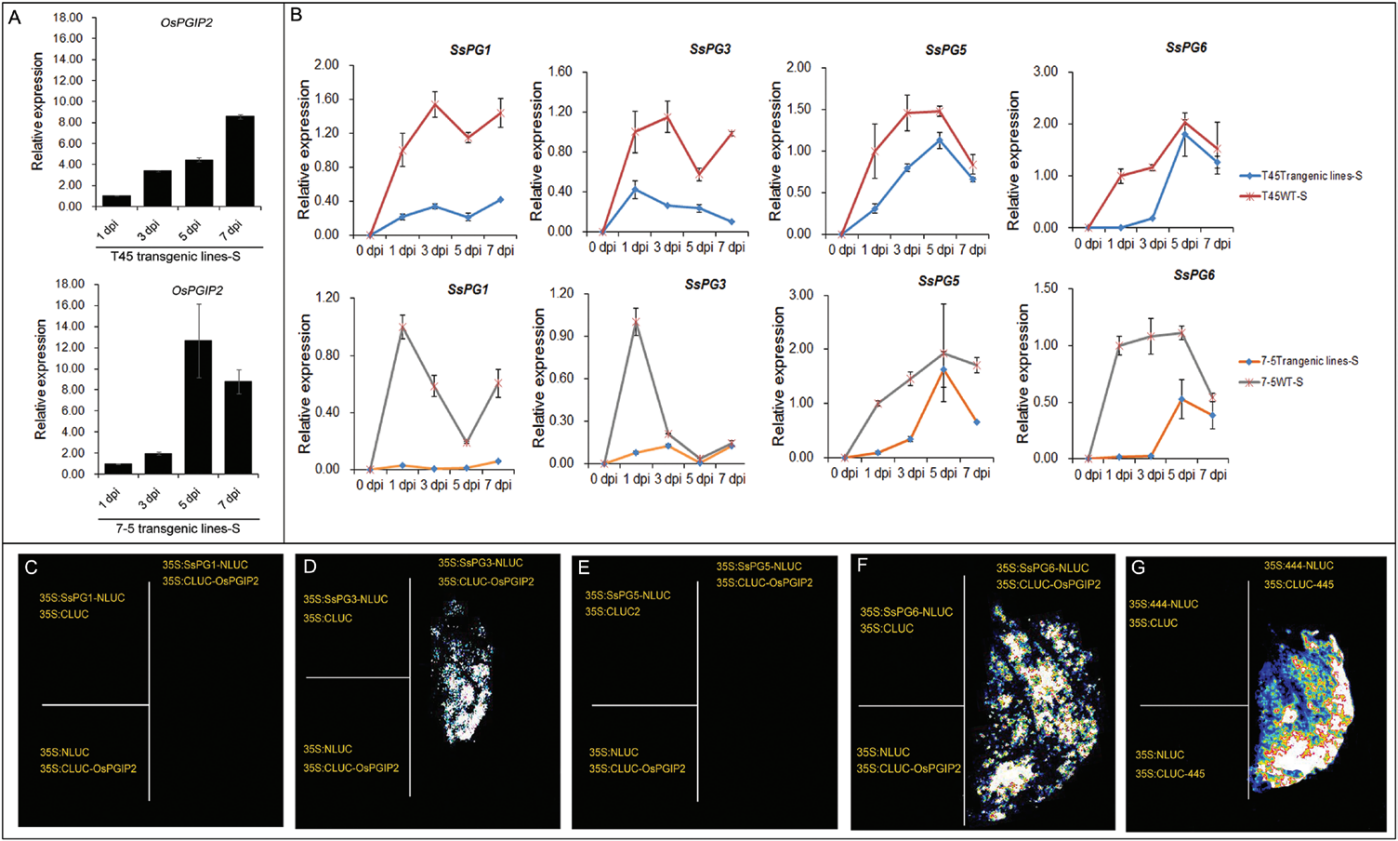
OsPGIP2 interacts with SsPG3 and SsPG6 when transiently expressed in *N. benthamiana* leaves. (A) The expression levels of *OsPGIP2* in transgenic and wild-type (WT) lines inoculated with *S. sclerotiorum*. qPCR results were normalized to *BnACTIN7* and *UBC21*. Data are means (±SD) and are representative of three biological and three technical replicates. (B) Expression levels of *S. sclerotiorum SsPG*s in transgenic and WT plants after inoculation. qPCR results were normalized to *S. sclerotiorum* 28S ribosomal DNA and histone *H3*. Three biological and three technical replicates were used in this experiment. (C–G) Transient expression assays to examine the interaction between OsPGIP2 and SsPGs. OsPGIP2 did not interact with SsPG1 (C); it interacted with SsPG3 (D); it did not interact with SsPG5 (E); it interacted with SsPG6 (F); positive control (G). Two biological replicates and two technical replicates were used in this experiment. The results of the interactions are presented on the right of each panel. The left of each panel shows the interaction between C-terminal luciferase (LUC) and the N-terminal target gene (top), and between the N-terminal LUC and the C-terminal target gene (bottom).

To further understand the mechanism of *S. sclerotiorum* resistance by *OsPGIP2* gene regulation, we measured gene expression in the stems of plants infected with *S. sclerotiorum* by qPCR. In 7-5 transgenic lines, the expression of *OsPGIP2* peaked at 5 dpi and then decreased; however, in T45 transgenic lines, the expression of *OsPGIP2* gradually increased over time (Fig. 5A). Furthermore, we found that the relative expression of the *S. sclerotiorum SsPG* genes changed at the necrotic site of the infected *B. napus*. The expressions of *SsPG1*, *SsPG3*, *SsPG5*, and *SsPG6* were significantly lower in the transgenic than in the wild-type lines (Fig. 5B). These results indicated that the enhanced *S. sclerotiorum* resistance in transgenic *OsPGIP2* lines might be due to a mechanism that reduces the expression of *S. sclerotiorum* pathogenic genes.

The expression of *S. sclerotiorum SsPG*s has been associated with the establishment and development of infection (Li *et al.*, 2004). Based on the hypothesis of a ‘PG–PGIP’ interaction (Benedetti *et al.*, 2011; Gutierrez, 2012), we hypothesized that OsPGIP2 could improve SSR resistance in *B. napus* by interacting with the PGs secreted by *S. sclerotiorum*. To examine this hypothesis, the affinities between OsPGIP2 and SsPGs were predicted using PPA-Pred2. The results showed that the affinities were higher in OsPGIP2–SsPG3 and OsPGIP2–SsPG6 than in OsPGIP2–SsPGl and OsPGIP2–SsPG5, suggesting that OsPGIP2 interacts more easily with SsPG3 and SsPG6 (see Supplementary Table S3). We cloned *OsPGIP2* into the C-terminus and *SsPG*s into the N-terminus of the luciferase reporter gene, and then co-transfected into *N. benthamiana* leaves using an *A. tumefaciens*-mediated transient expression system. Fluorescence observations indicated that SsPG3 and SsPG6 interacted with OsPGIP2, but SsPG1 and SsPG5 did not (Fig. 5C–G), indicating that OsPGIP2 might interact with SsPG3 and SsPG6 *in vivo* to enhance *S. sclerotiorum* resistance.

### Changes in cell wall monosaccharide content in the stems of *OsPGIP2*-expressing rapeseed

Plant cell walls largely consist of the polysaccharides cellulose, hemicellulose, pectin, and lignin. The cell wall structural profile was found to be altered during the development of transgenic *VvPGIP1* tobacco leaves (Nguema-Ona *et al.*, 2013). In our study, RNA-seq data showed that some cell wall synthetic and degrading enzymes were up-regulated in the transgenic *OsPGIP2* lines. Thus, we hypothesized that *OsPGIP2* overexpression might affect the composition of plant cell walls, and hence we determined the monosaccharide composition of rapeseed stem cell walls by HPLC (Table 3, and see Supplementary Fig. S8). The hemicellulose side-chain substitution rate (xylose/arabinose, Xyl/Ara) is also included in Table 3. Interestingly, although arabinose is not the major side-chain substitution for xylan in dicot plants, the xylose content of *OsPGIP2*-expressing lines in the flowering period was significantly lower than that of wild-type controls (Table 3). However, with flower development, the content of xylose dramatically increased in *OsPGIP2*-expressing lines compared to the wild-type. In 7-5WT and T45WT, the arabinose content from the flowering to final flowering stages gradually decreased. In contrast, arabinose contents gradually increased in the 7-5 and T45 transgenic lines during these developmental stages. The content of cellulose was evaluated based on the glucose content, and we found that transgenic lines had higher glucose and hemicellulose contents than the wild-type lines. Because lignin has an important role in cell wall remodeling, its content was further examined. As flowering progressed, the content of lignin in the stem increased in all the tested lines. In addition, except for T45B#1 and T45B#2, there were no significant differences in lignin content between the transgenic and control lines in the two flowering stages, indicating that overexpressing *OsPGIP2* had a low effect on lignin content (Table 3).

**Table 3.** Cell wall composition profile (%, mass ratio) of OsPGIP2-overexpressing rapeseed stem samples

	Glu	Xyl	Gal	Ara	Man	Xyl/Ara	Hemicellulose	Total lignin
Flowering period								
7-5WT	27.47 ± 0.17	10.58 ± 0.09	0.0531 ± 0.0004	1.84 ± 0.04	2.06 ± 0.04	5.78 ± 0.18	14.51 ± 0.03	11.46 ± 0.04
7-5C	30.46 ± 0.29**	6.96 ± 0.09**	0.0535 ± 0.0009	1.81 ± 0.10	2.79 ± 0.07*	3.86 ± 0.17**	11.61 ± 0.26**	11.14 ± 0.63
7-5D	30.35 ± 0.23**	7.66 ± 0.06**	0.0519 ± 0.0011	1.65 ± 0.27	2.86 ± 0.11*	4.65 ± 0.22*	12.21 ± 0.12*	12.59 ± 0.75
7-5G	30.48 ± 0.38**	7.08 ± 0.05**	0.0584 ± 0.0087	1.55 ± 0.25	2.83 ± 0.16*	4.60 ± 0.10*	11.51 ± 0.08**	13.13 ± 0.11
T45WT	26.91 ± 0.27	7.76 ± 0.06	0.0540 ± 0.0047	1.83 ± 0.01	1.88 ± 0.10	4.25 ± 0.02	11.52 ± 0.05	14.51 ± 0.13
T45B#1	27.33 ± 0.28	6.37 ± 0.11*	0.0480 ± 0.0047	1.65 ± 0.04	2.05 ± 0.53	3.87 ± 0.15*	10.11 ± 0.06*	15.91 ± 0.47
T45B#2	27.62 ± 0.33	4.91 ± 0.24**	0.0528 ± 0.0016	1.72 ± 0.03	2.20 ± 0.03**	2.04 ± 0.09**	8.88 ± 0.24**	13.05 ± 0.86
T45C	27.69 ± 0.15	5.47 ± 0.14**	0.0550 ± 0.0028	1.79 ± 0.13	2.07 ± 0.44	3.04 ± 0.12**	9.39 ± 0.11*	13.24 ± 0.50
Final flowering period								
7-5WT	30.39 ± 0.95	10.97 ± 0.77	0.0517 ± 0.0015	1.63 ± 0.04	2.56 ± 0.13	6.78 ± 0.61	15.21 ± 0.62	18.37 ± 0.04
7-5C	32.51 ± 0.28**	13.25 ± 0.06**	0.0510 ± 0.0009	1.85 ± 0.05	3.05 ± 0.02*	7.17 ± 0.19**	18.20 ± 0.05**	20.03 ± 0.10
7-5D	31.65 ± 0.64*	11.39 ± 0.03	0.0530 ± 0.0021	1.72 ± 0.03	3.29 ± 0.12**	6.64 ± 0.11*	16.43 ± 0.13*	20.57 ± 0.53
7-5G	31.32 ± 0.22*	14.12 ± 0.14**	0.0521 ± 0.0013	2.53 ± 0.05**	3.19 ± 0.09**	5.60 ± 0.05*	19.89 ± 0.25***	20.91 ± 0.05
T45WT	31.74 ± 0.11	8.47 ± 0.17	0.0524 ± 0.0011	0.66 ± 0.13	1.91 ± 0.10	12.91 ± 0.27	11.09 ± 0.12	17.28 ± 0.22
T45B#1	32.92 ± 0.78*	11.67 ± 0.13**	0.0532 ± 0.0025	2.48 ± 0.27**	2.80 ± 0.06**	4.71 ± 0.18**	17.00 ± 0.04***	20.10 ± 0.29*
T45B#2	32.67 ± 0.24*	10.65 ± 0.18**	0.0547 ± 0.0015	2.19 ± 0.11**	2.59 ± 0.05*	4.88 ± 0.10**	15.69 ± 0.46**	22.30 ± 0.14**
T45C	32.96 ± 0.71*	10.43 ± 0.20**	0.0554 ± 0.0033	2.08 ± 0.10**	3.00 ± 0.12***	5.04 ± 0.04**	15.56 ± 0.18**	19.84 ± 1.24

Glu, glucose; Xyl, xylose; Gal, galactose; Ara, arabinose; Man, mannose. Total lignin includes acid-insoluble and acid-soluble lignin. Analyses were performed using three biological replicates for wild-type and transgenic plants. Data are means ±SD. Significance was tested by one-way ANOVA: ***P<0.001, **P<0.01, *P<0.05. 7-5WT and T45WT are the wild-type controls.

## Discussion

The resistance of rapeseed to *S. sclerotiorum* obtained by using transgenic technology has mainly been based on observations of resistance in detached leaves in *in vitro* experiments (Wang *et al.*, 2014; Liu *et al.*, 2015). In the present study, transgenic *OsPGIP2*-overexpressing lines were created in two *B. napus* backgrounds and resulted in rapeseed plants with increased resistance to *S. sclerotiorum*, as indicated by survival tests after spraying with fungal hyphae and by the assessment of three consecutive years of stem inoculation with *S. sclerotiorum* in the field. Because transgenic *OsPGIP2* lines showed significantly smaller lesions than wild-type plants in detached-leaf inoculation experiments at 72 but not at 48 hpi, the function of *OsPGIP2* in increasing SSR resistance might have its major effect during the expansion of the infection. Thus, RNA-seq of transgenic *B. napus* was performed at 3 d after *S. sclerotiorum* infection.

When plants are infected with *S. sclerotiorum*, they show lower 1000-seed weight and lower seed quality than non-infected plants in soybean (Danielson *et al.*, 2004). Although we did not focus on the yield difference between the transgenic and wild-type lines, we also detected a reduction in the 1000-seed weight and significant effects on seed quality traits after *S. sclerotiorum* inoculation. Lower expression of GSL biosynthesis genes was related to lower GSL contents in the 7-5 and T45 transgenic lines after inoculation (see Supplementary Fig. S9A and Supplementary File S3). However, Wu *et al.* (2016) compared gene transcription between resistant and susceptible *B. napu*s lines inoculated with *S. sclerotiorum* and found that biosynthesis of indolic GSL was more intensely induced in the resistant line than in the susceptible line, and these results are consistent with the expression of indolic GSL biosynthesis genes. Zhao and Meng (2003) found that the contents of aliphatic GSL and 3-indolyl-methyl GSL in seeds were in a positive relationship with *S. sclerotiorum* resistance on leaves at the seedling stage and on stems of maturing plants, respectively. In our present study, when comparing between low disease pressure and field stem inoculation, the wild-type seeds had significant changes in the content of GSL but this was not the case in the transgenic lines (Supplementary Fig. S9B). This means that the transgenic *OsPGIP2* rapeseed lines did not show additional induction of GSL production against SSR, indicating that *OsPGIP2* led to *S. sclerotiorum* resistance in transgenic lines through a mechanism that did not involve accumulation of GSL. Thus, there seems to be no direct correlation between disease resistance due to the *OsPGIP2* gene and synthesis of GSLs.

The ‘PG–PGIP’ interaction plays a key role in determining protein–protein recognition in plant–pathogen interactions. The pathogenic *PG*s from *S. sclerotiorum* are critical to both the establishment and expansion of infection (Li *et al.*, 2004). Bashi *et al.* (2013) demonstrated that BnPGIP1 could interact with SsPG6 *in vitro*, and, in the present study, we showed that OsPGIP2 could interact with SsPG3 and SsPG6 but not with SsPG1 and SsPG5 *in vivo*, indicating that the inhibitory activity of every PGIP is specific to a range of PGs from different fungal pathogens. For instance, the purified PGIP1 of *Malus domestica* inhibits the PGs from *Colletotrichum lupine*, *Botryosphaeria obtusa*, and *Diaporthe ambigua*, but not the *A. niger* PG (Oelofse *et al.*, 2006); PvPGIP1, PvPGIP2, and PvPGIP4 inhibit a PG from *A. niger* (Leckie *et al.*, 1999; D’Ovidio *et al.*, 2004) but PvPGIP3 does not (D’Ovidio *et al.*, 2004). However, *PvPGIP2*-expressing wheat lines infected with *Claviceps purpurea* do not show a clear reduction of disease symptoms due to the lack of function of inhibiting PGs from *C. purpurea* (Volpi *et al.*, 2013). Although the overexpression of *OsPGIP2* significantly increased *S. sclerotiorum* resistance in transgenic *B. napus* lines compared with wild-type lines, their resistance did not increase to the level shown by ZS11, indicating that increasing the expression of *PGIP* alone is not sufficient to greatly increase SSR resistance in rapeseed. In particular, *S. sclerotiorum* has evolved a variety of pathogenic factors, such as oxalic acid (OA), cerato-platanin protein 1 (CP1), SsCUTA (cutinase), and fungal integrin-like protein (SsITL) (Heard *et al.*, 2015; Yang *et al.*, 2018). Thus, although overexpressing *OsPGIP2* in rapeseed must involve the ‘PG–PGIP’ interaction, it is also necessary to find additional mechanisms of interaction between rapeseed and *S. sclerotiorum*, and to combine *PGIP* with other defense-response genes, such as PR-1 that interacts with SsCP1 in the apoplast (Yang *et al.*, 2018), in order to improve the degree of rapeseed resistance (Bashi *et al.*, 2013).

Biochemical and RNA-seq analyses of *OsPGIP2*-expressing rapeseed lines revealed that the content of cell wall monosaccharides and polysaccharide synthases or hydrolases were specifically up-regulated in the transgenic lines. Endotransglucosylases/hydrolases (XTHs) are believed to be important for regulating cell wall strength, extensibility, and tissue integrity (Saladié *et al.*, 2006). Cellulose synthases (CESAs) are involved in the synthesis of primary and secondary cell wall cellulose (Chundawat *et al.*, 2011). In tobacco, *VvPGIP1* overexpression results in the down-regulation of a group of XTHs and in a reduction of XTH enzyme activity in uninfected transgenic tobacco (Alexandersson *et al.*, 2011). However, in our present study, the cytoskeletal genes *CESA6* and *XTH24* were specifically up-regulated in the transgenic *OsPGIP2* lines, which had higher cellulose contents than the wild-type lines, indicating that *OsPGIP2* has a direct or indirect effect on cell wall modification, possibly leading to changes in cell wall monosaccharide metabolism. In addition, during the process of infection in plants, intracellular soluble sugar provides a source of nutrition for colonization by the pathogens (Doidy *et al.*, 2012). Thus, the enhancement of the metabolism and transport of carbon compounds in the transgenic *OsPGIP2* lines also demonstrated competition for nutrients between the plant and pathogen. Indeed, increased invertase activity and plant monosaccharide-importer expression may result in pathogens having reduced access to sugars during infection (Sutton *et al.*, 2007).

Reactive oxygen species are important mediators in PAMP-triggered immunity (PTI), contributing to defense responses during plant–pathogen interactions (Baxter *et al.*, 2014). The defense response is mediated by oxidative waves of ROS that activate signal transduction through phosphorylation cascades, accompanied by hormonal signaling and by the expression of defense-related genes (Baxter *et al.*, 2014). In *A. thaliana*, peroxidases have been identified as major contributors to ROS production during responses to fungal elicitors (Daudi *et al.*, 2012). In our present study, elevated H_2_O_2_ accumulation and expression of peroxidases were detected in the leaves of *OsPGIP2*-expressing lines after inoculation. Although ROS plays a role in resistance during early infection, more H_2_O_2_ may promote disease during later infection stages of *S. sclerotiorum* (Williams *et al.*, 2011). Therefore, to reduce H_2_O_2_ toxicity to plants, *CAT1*s and glutathione metabolic processes have been induced in transgenic lines, and this induction at the core of the plant antioxidant system protects ROS signaling and contributes to the removal of O_2_^–^ to protect the plant from developing lesions due to ROS toxicity (Foyer and Noctor, 2011).

**Fig. 6.**
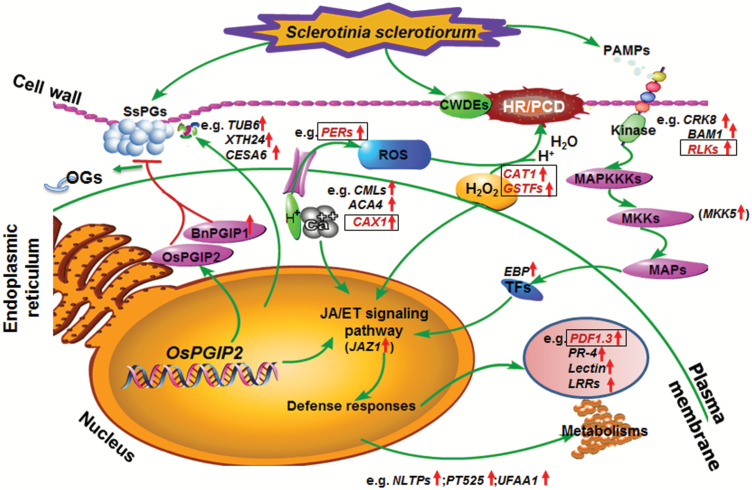
A model for the molecular mechanism by which *OsPGIP2* confers *S. sclerotiorum* resistance through increased activation of defense mechanisms. Upward arrows next to gene names highlight the up-regulation of differentially expressed genes, as revealed in RNA-seq analysis. Genes highlighted in boxes are the common up-regulated DEGs in the 7-5 and T45 backgrounds. Gene details are given in Supplementary File S2. CWDEs, cell wall-degrading enzymes; HR/PCD, hypersensitive response/programmed cell death; MAPs, mitogen-activated proteins; MAPKKKs, MAP-kinase-kinase kinases; MKKs, MAP-kinase kinases; OGs, oligogalacturonides; PAMPs, pathogen-associated molecular patterns; PGIP, polygalacturonase inhibitor protein; ROS, reactive oxygen species; SsPGs, *S. sclerotiorum* polygalacturonases; TFs, transcription factors.

Although the mechanism by which *OsPGIP2* reduces SSR development is still largely unknown, the RNA-seq results presented here hint at a possible molecular mechanism. Based on changes in gene expression in response to infection in the transgenic rapeseed lines, we propose a model for *OsPGIP2*-conferred resistance to *S. sclerotiorum* in these plants (Fig. 6). After *S. sclerotiorum* infection, PAMP perception and a series of signal transductions are initiated, and then followed by the activation of several *B. napus* defenses aimed at delaying *S. sclerotiorum* development. These include: (i) increasing ROS (H_2_O_2_-derived) to active the innate immune pathway (Fig. 4); (ii) reinforcing cell walls to inhibit the growth of *S. sclerotiorum* (Table 3); (iii) increasing the accumulation of OsPGIP2 to inhibit SsPG3 and SsPG6 (Fig. 5); and increasing the accumulation of BnPGIP1 to inhibit SsPG6 (Bashi *et al.*, 2013). As a result of the synergistic actions of these genes, the transgenic rapeseed plants have a stronger resistance to *S. sclerotiorum* than wild-type plants at both the seedling and adult stages. In summary, the increased resistance observed in transgenic *OsPGIP2*-expressing lines suggests that overexpression can activate the basal defense pathway, which provides a molecular basis for the application of *OsPGIP2* to develop plants resistant to fungal pathogens in other important crops.

## Supplementary data

Supplementary data are available at *JXB* online.

Fig. S1. Analysis of *OsPGIP2* gene expression in transgenic lines by molecular identification methods.

Fig. S2. The phenotypes of seedlings and adult plants of T_4_ transgenic and wild-type lines.

Fig. S3. Seed quality traits of T_4_ transgenic lines following inoculation with *S. sclerotiorum*.

Fig. S4. Clustered heatmap and GO enrichment analysis of differentially expressed genes between transgenic and wild-type lines of *B. napus* responsive to *S. sclerotiorum*.

Fig. S5. Clustered heatmap analysis of down-regulated differentially expressed genes between transgenic and wild-type lines of *B. napus* responsive to *S. sclerotiorum*.

Fig. S6. qPCR confirmation of the differentially expressed genes between 7-5 transgenic and wild-type lines after inoculation.

Fig. S7. qPCR confirmation of the differentially expressed genes between T45 transgenic and wild-type lines after inoculation.

Fig. S8. Determination of cell wall monosaccharides in the transgenic lines at different stages by HPLC.

Fig. S9. Expression of GSL biosynthesis genes and the seed content of GSL.

Table S1. Field testing data for disease severity for individual rapeseed lines in three consecutive years.

Table S2. Number of clean sequence reads that mapped to the *S. sclerotiorum* 1980 genome.

Table S3. The affinity between OsPGIP2 and *S. sclerotiorum* PGs.

Table S4. List of primers used in this study.

File S1. List of all differentially expressed gene annotations and FPKM values.

File S2. Up- and down-regulated differentially expressed genes in defense related pathways.

File S3. Transcript abundance of GSL genes for transgenic and wild-type lines.

Supplemental Figures and Tables

Supplementary Dataset S1

Supplementary Dataset S2

Supplementary Dataset S3
