# Supplementary material for: Overexpression of OsPGIP2 confers Sclerotinia sclerotiorum resistance in Brassica napus through increased activation of defense mechanisms
Source: J Exp Bot. 2018 Apr 10;69(12):3141–55. doi: 10.1093/jxb/ery138 (PMC5972623; doi:10.1093/jxb/ery138)
Supplement: Supplemental Figures and Tables [file ery138_suppl_supplemental_figures_and_tables.pdf]

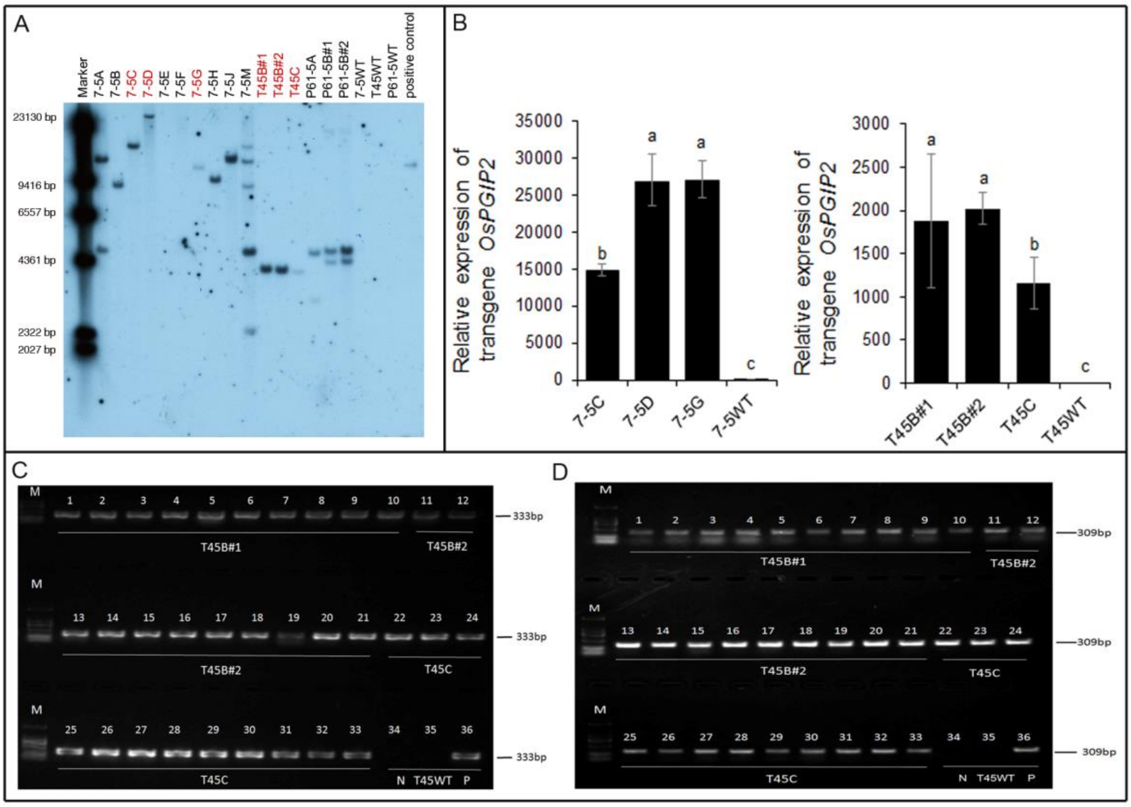

Fig. S1. Analysis of *OsPGIP2* gene expression in transgenic lines by molecular identification methods.

(A) Southern blot analysis of the copy number of transgenic lines overexpressing *OsPGIP2* gene. 50 μg of genomic DNA was digested with *KpnI* for southern blot detection. 7-5A, 7-5B, 7-5C, 7-5D, 7-5E, 7-5F, 7-5G, 7-5H, 7-5J, 7-5M, T45B#1, T45B#2, T45C, P61-5A, P61-5B#1 and P61-5B#2 are transgenic rapeseed lines. 7-5WT, T45WT and P61-5WT are non-transgenic rapeseed controls. Positive control is pCAMIBA1300-35S:*OsPGIP2* plasmid. Red indicates the transgenic lines selected for further study. (B) The expression level of *OsPGIP2* gene in T_2 homozygous transgenic plant lines. In transgenic plant lines 7-5C, 7-5D, 7-5G, T45-B#1, T45B#2 and T45C, the relative level of *OsPGIP2* expression was normalized to the rapeseed *BnACTIN7* gene (*AF111812*). Bars represent mean values from three biological replicates ± standard deviation. Different letters indicate values that are significantly different at $P < 0.05$ as tested by least significant difference (LSD) multiple-comparison test. Non-transgenic lines 7-5WT and T45WT served as controls, respectively. (C) and (D)

analysis of T_4 transgenic lines by polymerase chain reaction (representative results). Tested the *OsPGIP2* gene in T45 transgenic lines with 333-L/R primer (C); tested the *HYG* gene in T45 transgenic lines with 309-*HYG*-L/R primer (D). M refers to DL2000 marker, T45WT is non-transgenic lines control. N means ddH_2O as negative control. P is plasmid DNA as positive control.

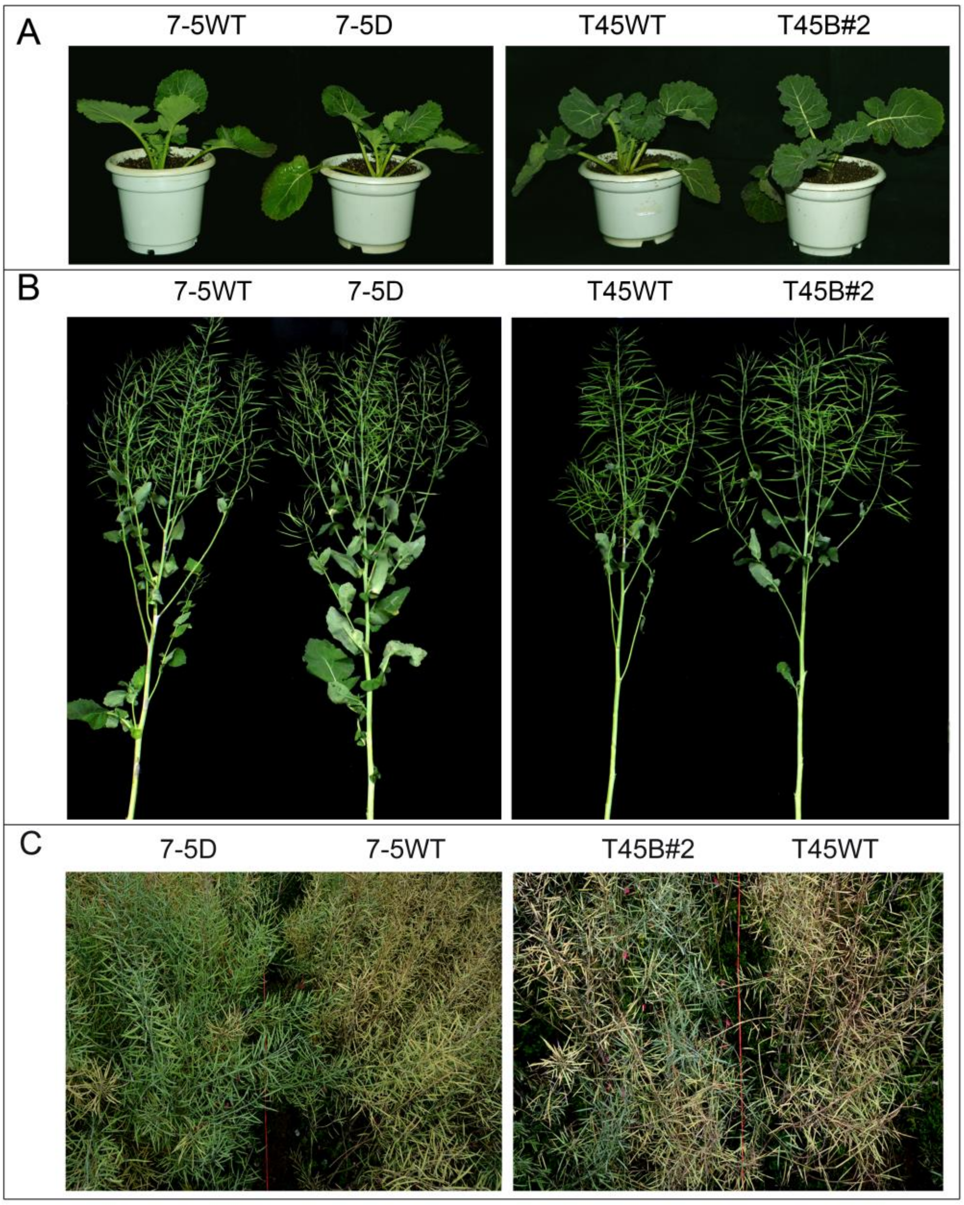

Fig. S2. The phenotypes of seedling and adult plant of T_4 transgenic and wild type lines.

The phenotypes of 13-week-old seedlings (A) and the plants at the stage of silique maturity (B) without inoculation with *S. sclerotiorum*. Sprayed mycelium within PDB liquid media on adult plants of T_4 transgenic and wild type lines (C)**.** At the flowering period, transgenic lines and control *B. napus* plants were sprayed with mycelium suspension (50%). Infection phenotypes were assessed at 30 dpi. 7-5WT and T45WT are non-transgenic controls.

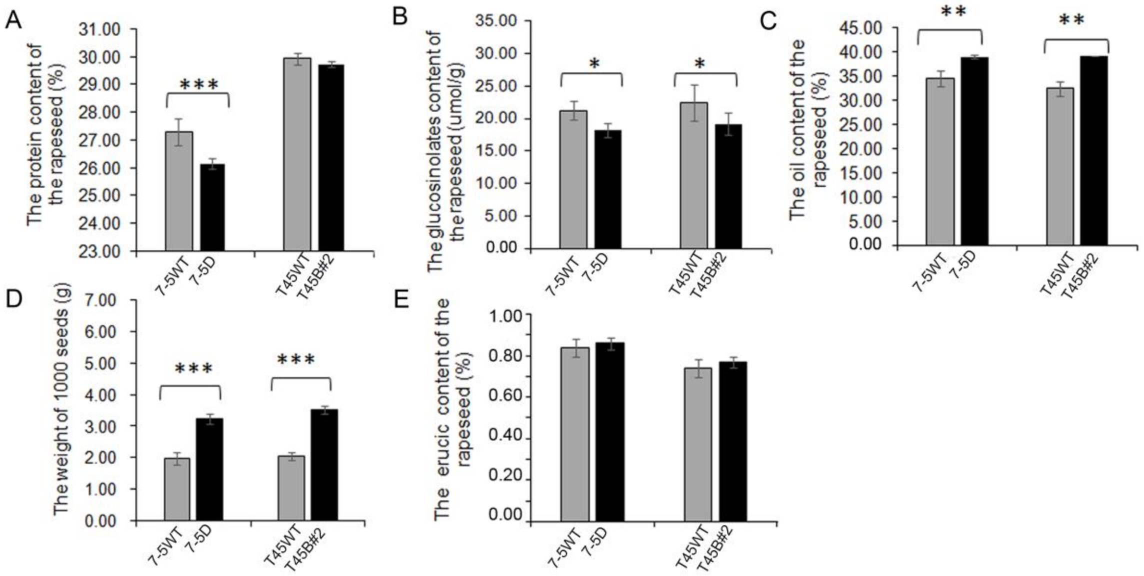

Fig. S3. Seed quality traits of T_4 transgenic lines in the isolated field under the mycelia inoculation with *S. sclerotiorum*.

The protein content (A), glucosinolate content (B), oil content (C), 1000-seed weight (D) and erucic content (E) were measured based on dry condition. Values are means of six replicates from two randomized complete blocks ± standard deviation. One-way ANOVA was used to test statistical significance. Bars with one asterisk was significant difference at $P < 0.05$, two asterisks were significant difference at $P < 0.01$ and three asterisks were significant difference at $P < 0.001$.

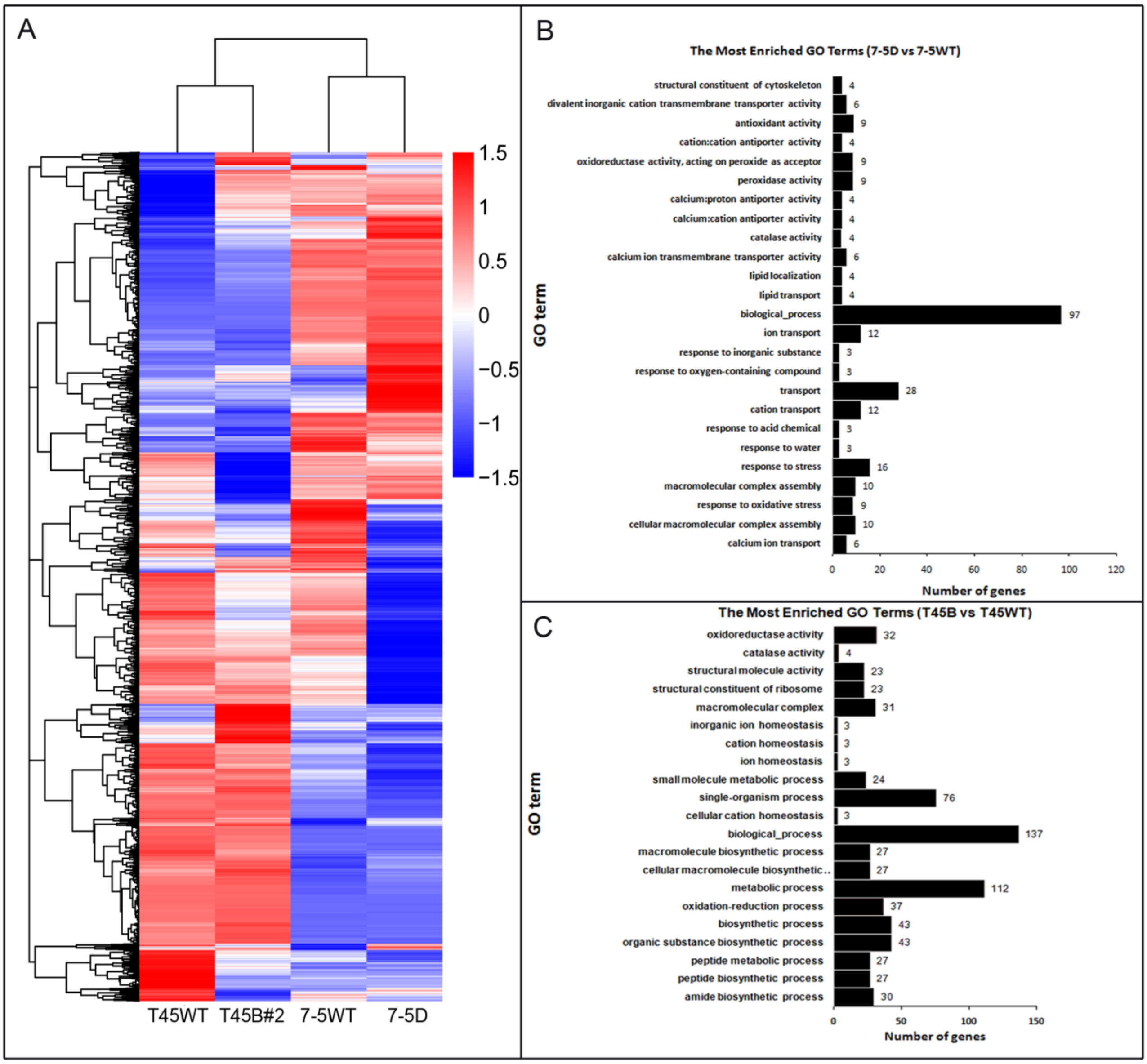

Fig. S4. Clustered heatmap and GO enrichment analysis of differentially expressed genes (DEGs) of *B. napus* genes responsive to *S. sclerotiorum* between transgenic and wild type lines.

(A) All differential genes expression (T45B#2 vs T45WT (605), 7-5D vs 7-5WT (886)) were clustered in the heatmap of based on FPKM (fragments per kilobase of exon per million fragments mapped) levels in transgenic *B. napus* and wild type lines in response to *S. sclerotiorum* infection. The DEGs are clustered on the Y axis according to hierarchical agglomerative clustering. The FPKM was normalized using the log_{10} $(fpkm+1)$ to generate the heatmap. Red and blue represent high and low gene expression, respectively. The gene details are shown in the File S1. Upregulated differentially

expressed genes (up-DEGs) were analyzed by gene ontology (GO) enrichment in T45B#2 vs. T45WT (B) and 7-5D vs. 7-5WT responsive to *S. sclerotiorum* (C). The software method used in the GO enrichment analysis was GOseq (Young et al, 2010) based on the Wallenius non-central hyper-geometric distribution. Gene Ontology lists of Arabidopsis(ftp://ftp.ncbi.nlm.nih.gov/blast/db/FASTA/) were used.

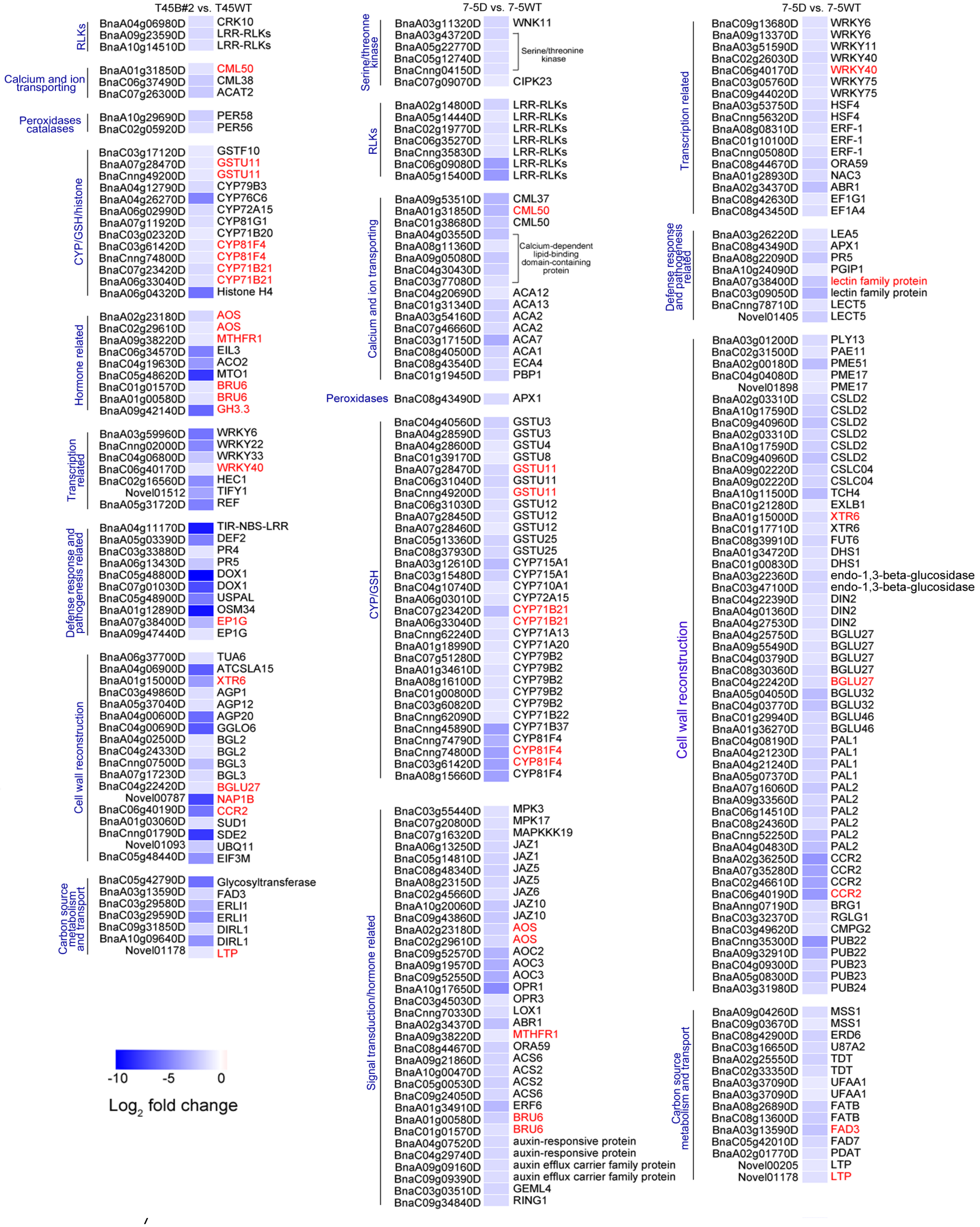

T45B#2 vs. T45WT
RLKs
BnaA04g06980D CRK10
BnaA09g23590D LRR-RLKs
BnaA10g14510D LRR-RLKs
Calcium and ion transporting
BnaA01g31850D CML50
BnaC06g37490D CML38
BnaC07g26300D ACAT2
Peroxidases catalases
BnaA10g29690D PER58
BnaC02g05920D PER56
CYP/GSH/histone
BnaC03g17120D GSTF10
BnaA07g28470D GSTU11
BnaCnng49200D GSTU11
BnaA04g12790D CYP79B3
BnaA04g26270D CYP76C6
BnaA06g02990D CYP72A15
BnaA07g11920D CYP81G1
BnaC03g02320D CYP71B20
BnaC03g61420D CYP81F4
BnaCnng74800D CYP81F4
BnaC07g23420D CYP71B21
BnaA06g33040D CYP71B21
BnaA06g04320D Histone H4
Hormone related
BnaA02g23180D AOS
BnaC02g29610D AOS
BnaA09g38220D MTHFR1
BnaC06g34570D EIL3
BnaC04g19630D ACO2
BnaC05g48620D MTO1
BnaC01g01570D BRU6
BnaA01g00580D BRU6
BnaA09g42140D GH3.3
Transcription related
BnaA03g59960D WRKY6
BnaCnng02000D WRKY22
BnaC04g06800D WRKY33
BnaC06g40170D WRKY40
BnaC02g16560D HEC1
Novel01512 TIFY1
BnaA05g31720D REF
Defense response and pathogenesis related
BnaA04g11170D TIR-NBS-LRR
BnaA05g03390D DEF2
BnaC03g33880D PR4
BnaA06g13430D PR5
BnaC05g48800D DOX1
BnaC07g01030D DOX1
BnaC05g48900D USPAL
BnaA01g12890D OSM34
BnaA07g38400D EP1G
BnaA09g47440D EP1G
Cell wall reconstruction
BnaA06g37700D TUA6
BnaA04g06900D ATCSLA15
BnaA01g15000D XTR6
BnaC03g49860D AGP1
BnaA05g37040D AGP12
BnaA04g00600D AGP20
BnaC04g00690D GGLO6
BnaA04g02500D BGL2
BnaC04g24330D BGL2
BnaCnng07500D BGL3
BnaA07g17230D BGL3
BnaC04g22420D BGLU27
Novel00787 NAP1B
BnaC06g40190D CCR2
BnaA01g03060D SUD1
BnaCnng01790D SDE2
Novel01093 UBQ11
BnaC05g48440D EIF3M
Carbon source metabolism and transport
BnaC05g42790D Glycosyltransferase
BnaA03g13590D FAD3
BnaC03g29580D ERLI1
BnaC03g29590D ERLI1
BnaC09g31850D DIRL1
BnaA10g09640D DIRL1
Novel01178 LTP
-10 -5 0
Log$_2$ fold change
7-5D vs. 7-5WT
Serine/threonine kinase
BnaA03g11320D WNK11
BnaA03g43720D
BnaA05g22770D
BnaC05g12740D
BnaCnng04150D
Serine/threonine kinase
BnaC07g09070D CIPK23
RLKs
BnaA02g14800D LRR-RLKs
BnaA05g14440D LRR-RLKs
BnaC02g19770D LRR-RLKs
BnaC06g35270D LRR-RLKs
BnaCnng35830D LRR-RLKs
BnaC06g09080D LRR-RLKs
BnaA05g15400D LRR-RLKs
Calcium and ion transporting
BnaA09g53510D CML37
BnaA01g31850D CML50
BnaC01g38680D CML50
BnaA04g03550D
BnaA08g11360D
BnaA09g05080D
BnaC04g30430D
BnaC03g77080D
Calcium-dependent lipid-binding domain-containing protein
BnaC04g20690D ACA12
BnaC01g31340D ACA13
BnaA03g54160D ACA2
BnaC07g46660D ACA2
BnaC03g17150D ACA7
BnaC08g40500D ACA1
BnaC08g43540D ECA4
BnaC01g19450D PBP1
Peroxidases
BnaC08g43490D APX1
CYP/GSH
BnaC04g40560D GSTU3
BnaA04g28590D GSTU3
BnaA04g28600D GSTU4
BnaC01g39170D GSTU8
BnaA07g28470D GSTU11
BnaC06g31040D GSTU11
BnaCnng49200D GSTU11
BnaC06g31030D GSTU12
BnaA07g28450D GSTU12
BnaA07g28460D GSTU12
BnaC05g13360D GSTU25
BnaC08g37930D GSTU25
BnaA03g12610D CYP715A1
BnaC03g15480D CYP715A1
BnaC04g10740D CYP710A1
BnaA06g03010D CYP72A15
BnaC07g23420D CYP71B21
BnaA06g33040D CYP71B21
BnaCnng62240D CYP71A13
BnaA01g18990D CYP71A20
BnaC07g51280D CYP79B2
BnaA01g34610D CYP79B2
BnaA08g16100D CYP79B2
BnaC01g00800D CYP79B2
BnaC03g60820D CYP79B2
BnaCnng62090D CYP71B22
BnaCnng45890D CYP71B37
BnaCnng74790D CYP81F4
BnaCnng74800D CYP81F4
BnaC03g61420D CYP81F4
BnaA08g15660D CYP81F4
Signal transduction/hormone related
BnaC03g55440D MPK3
BnaC07g20800D MPK17
BnaC07g16320D MAPKKK19
BnaA06g13250D JAZ1
BnaC05g14810D JAZ1
BnaC08g48340D JAZ5
BnaA08g23150D JAZ5
BnaC02g45660D JAZ6
BnaA10g20060D JAZ10
BnaC09g43860D JAZ10
BnaA02g23180D AOS
BnaC02g29610D AOS
BnaC09g52570D AOC2
BnaA09g19570D AOC3
BnaC09g52550D AOC3
BnaA10g17650D OPR1
BnaC03g45030D OPR3
BnaCnng70330D LOX1
BnaA02g34370D ABR1
BnaA09g38220D MTHFR1
BnaC08g44670D ORA59
BnaA09g21860D ACS6
BnaA10g00470D ACS2
BnaC05g00530D ACS2
BnaC09g24050D ACS6
BnaA01g34910D ERF6
BnaA01g00580D BRU6
BnaC01g01570D BRU6
BnaA04g07520D auxin-responsive protein
BnaC04g29740D auxin-responsive protein
BnaA09g09160D auxin efflux carrier family protein
BnaC09g09390D auxin efflux carrier family protein
BnaC03g03510D GEML4
BnaC09g34840D RING1
7-5D vs. 7-5WT
Transcription related
BnaC09g13680D WRKY6
BnaA09g13370D WRKY6
BnaA03g51590D WRKY11
BnaC02g26030D WRKY40
BnaC06g40170D WRKY40
BnaC03g05760D WRKY75
BnaC09g44020D WRKY75
BnaA03g53750D HSF4
BnaCnng56320D HSF4
BnaA08g08310D ERF-1
BnaC01g10100D ERF-1
BnaCnng05080D ERF-1
BnaC08g44670D ORA59
BnaA01g28930D NAC3
BnaA02g34370D ABR1
BnaC08g42630D EF1G1
BnaC08g43450D EF1A4
Defense response and pathogenesis related
BnaA03g26220D LEA5
BnaC08g43490D APX1
BnaA08g22090D PR5
BnaA10g24090D PGIP1
BnaA07g38400D lectin family protein
BnaC03g09050D lectin family protein
BnaCnng78710D LECT5
Novel01405 LECT5
Cell wall reconstruction
BnaA03g01200D PLY13
BnaC02g31500D PAE11
BnaA02g00180D PME51
BnaC04g04080D PME17
Novel01898 PME17
BnaA02g03310D CSLD2
BnaA10g17590D CSLD2
BnaC09g40960D CSLD2
BnaA02g03310D CSLD2
BnaA10g17590D CSLD2
BnaC09g40960D CSLD2
BnaA09g02220D CSLC04
BnaA09g02220D CSLC04
BnaA10g11500D TCH4
BnaC01g21280D EXLB1
BnaA01g15000D XTR6
BnaC01g17710D XTR6
BnaC08g39910D FUT6
BnaA01g34720D DHS1
BnaC01g00830D DHS1
BnaA03g22360D endo-1,3-beta-glucosidase
BnaC03g47100D endo-1,3-beta-glucosidase
BnaC04g22390D DIN2
BnaA04g01360D DIN2
BnaA04g27530D DIN2
BnaA04g25750D BGLU27
BnaA09g55490D BGLU27
BnaC04g03790D BGLU27
BnaC08g30360D BGLU27
BnaC04g22420D BGLU27
BnaA05g04050D BGLU32
BnaC04g03770D BGLU32
BnaC01g29940D BGLU46
BnaA01g36270D BGLU46
BnaC04g08190D PAL1
BnaA04g21230D PAL1
BnaA04g21240D PAL1
BnaA05g07370D PAL1
BnaA07g16060D PAL2
BnaA09g33560D PAL2
BnaC06g14510D PAL2
BnaC08g24360D PAL2
BnaCnng52250D PAL2
BnaA04g04830D PAL2
BnaA02g36250D CCR2
BnaA07g35280D CCR2
BnaC02g46610D CCR2
BnaC06g40190D CCR2
BnaAnng07190D BRG1
BnaC03g32370D RGLG1
BnaC03g49620D CMPG2
BnaCnng35300D PUB22
BnaA09g32910D PUB22
BnaC04g09300D PUB23
BnaA05g08300D PUB23
BnaA03g31980D PUB24
Carbon source metabolism and transport
BnaA09g04260D MSS1
BnaC09g03670D MSS1
BnaC08g42900D ERD6
BnaC03g16650D U87A2
BnaA02g25550D TDT
BnaC02g33350D TDT
BnaA03g37090D UFAA1
BnaA03g37090D UFAA1
BnaA08g26890D FATB
BnaC08g13600D FATB
BnaA03g13590D FAD3
BnaC05g42010D FAD7
BnaA02g01770D PDAT
Novel00205 LTP
Novel01178 LTP

Fig. S5. Clustered heatmap analysis of down differentially expressed genes (down-DEGs) of *B. napus* genes responsive to *S. sclerotiorum* between transgenic and wild type lines.

Down-DEGs were considered statistically significant if qvalue < 0.005 and $|\log_2$Fold change$| > 1$. Blue in bar represent low gene expression, and genes marked in red means the common down-DEGs in 7-5 and T45 background. Gene details are given in File S2.

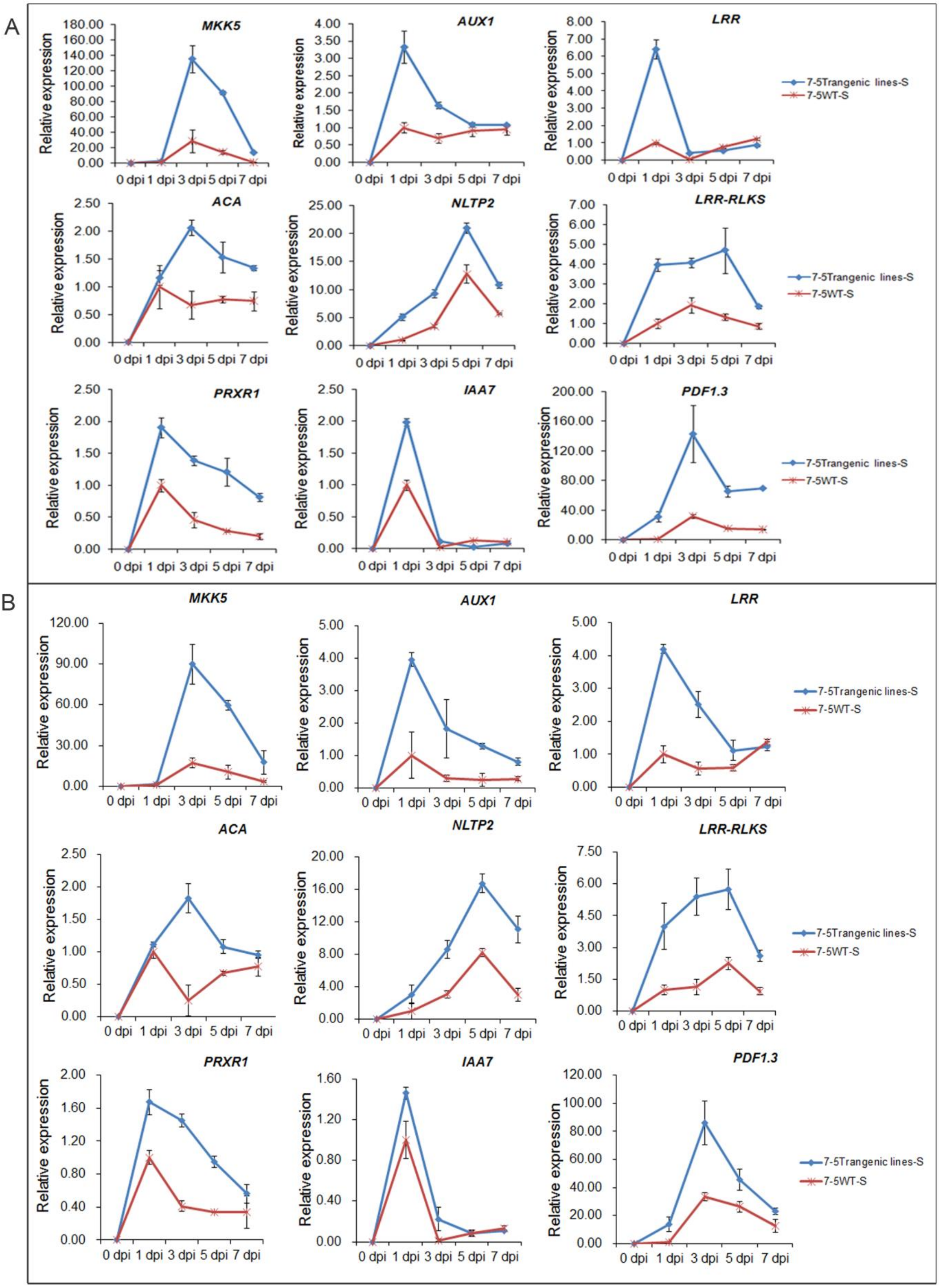

Fig. S6. qPCR confirmation of the differentially expressed genes (DEGs) between

7-5 transgenic lines and wild type lines after inoculation.

Total RNA was extracted from stem inoculated with *S. sclerotiorum* over time. The expression levels of these genes in transgenic and WT lines inoculated with *S. sclerotiorum* were determined by qPCR and normalized to *BnACTIN7* (A) and ubiquitin-conjugating enzyme 21 (B). Values are means ± SD (n = 3). Error bars represent standard deviation from three biological replicates. The gene details are shown in the File S2 and the primers are listed in the table S4.

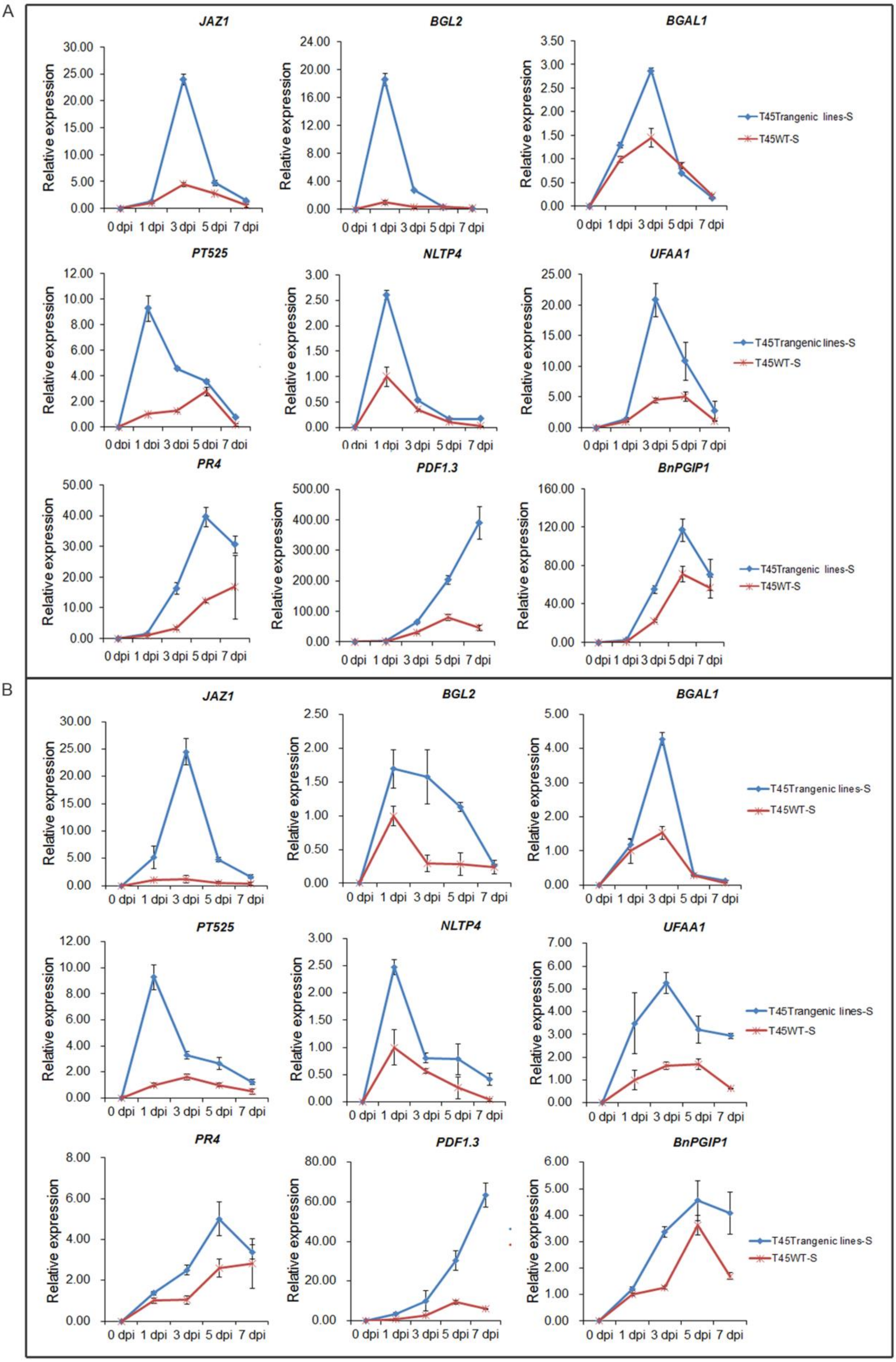
A
JAZ1
BGL2
BGAL1
PT525
NLTP4
UFAA1
PR4
PDF1.3
BnPGIP1
Relative expression
0 dpi 1 dpi 3 dpi 5 dpi 7 dpi
T45Trangenic lines-S
T45WT-S
B
JAZ1
BGL2
BGAL1
PT525
NLTP4
UFAA1
PR4
PDF1.3
BnPGIP1
Relative expression
0 dpi 1 dpi 3 dpi 5 dpi 7 dpi
T45Trangenic lines-S
T45WT-S

Fig. S7. qPCR confirmation of the differentially expressed genes (DEGs) between T45 transgenic lines and non-transgenic lines after inoculation.

Total RNA was extracted from stem inoculated with *S. sclerotiorum* over time. The expression levels of these genes in transgenic and WT lines inoculated with *S. sclerotiorum* were determined by qPCR and normalized to *BnACTIN7* (A) and ubiquitin-conjugating enzyme 21 (B). Values are means ± SD (n = 3). Error bars represent standard deviation from three biological replicates. The gene details are shown in the File S2 and the primers are listed in the table S4.

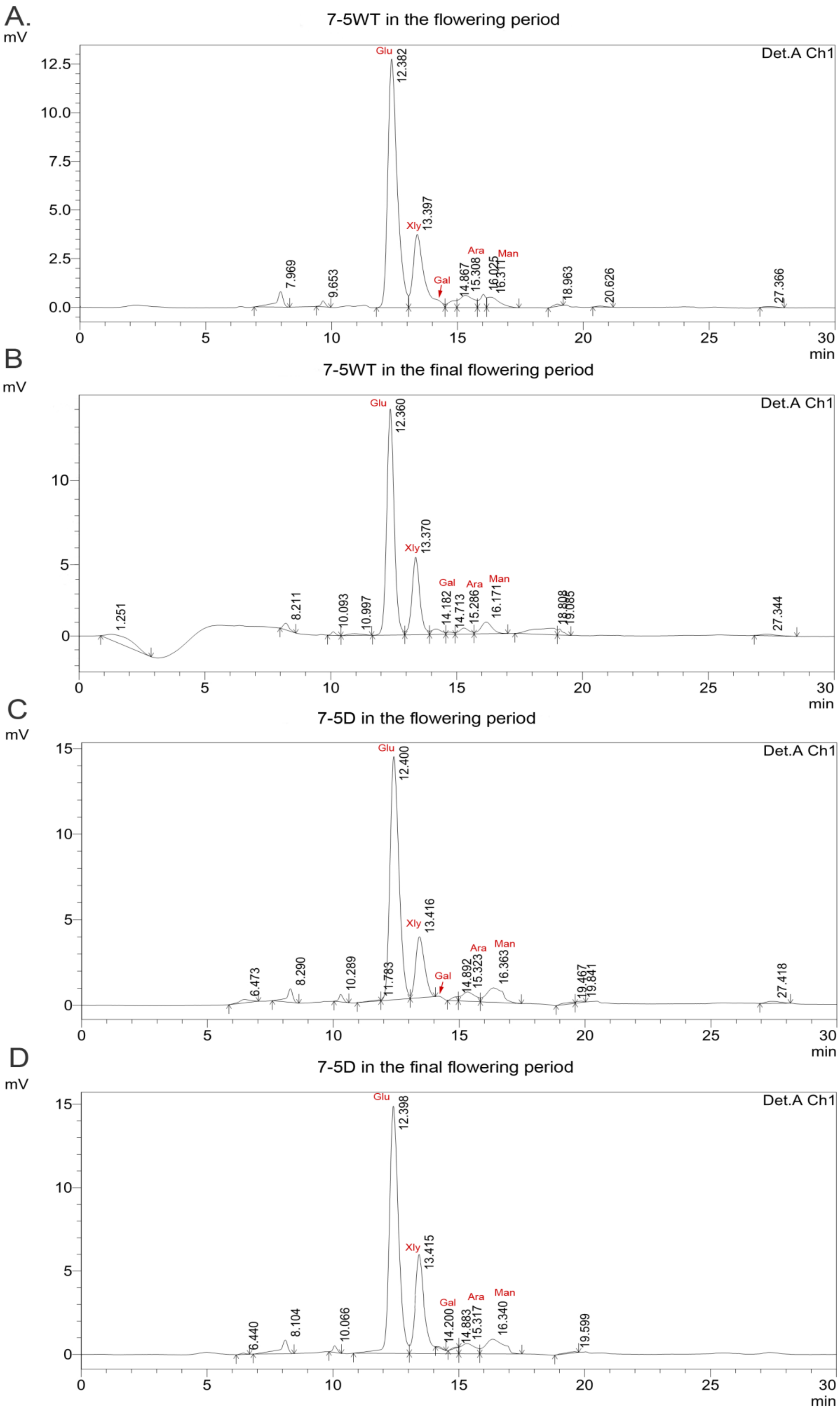
A.
7-5WT in the flowering period
mV
Det.A Ch1
Glu
12.382
Xly
13.397
Gal
Ara
Man
7.969
9.653
14.867
15.308
16.025
16.311
18.963
20.626
27.366
min
B
7-5WT in the final flowering period
mV
Det.A Ch1
Glu
12.360
Xly
13.370
Gal
Ara
Man
1.251
8.211
10.093
10.997
14.182
14.713
15.286
16.171
18.808
19.085
27.344
min
C
7-5D in the flowering period
mV
Det.A Ch1
Glu
12.400
Xly
13.416
Gal
Ara
Man
6.473
8.290
10.289
11.783
14.892
15.323
16.363
19.467
19.841
27.418
min
D
7-5D in the final flowering period
mV
Det.A Ch1
Glu
12.398
Xly
13.415
Gal
Ara
Man
6.440
8.104
10.066
14.200
14.883
15.317
16.340
19.599
min

Fig. S8. Determination of cell wall monosaccharide in the transgenic lines at different stages by high performance liquid chromatography.

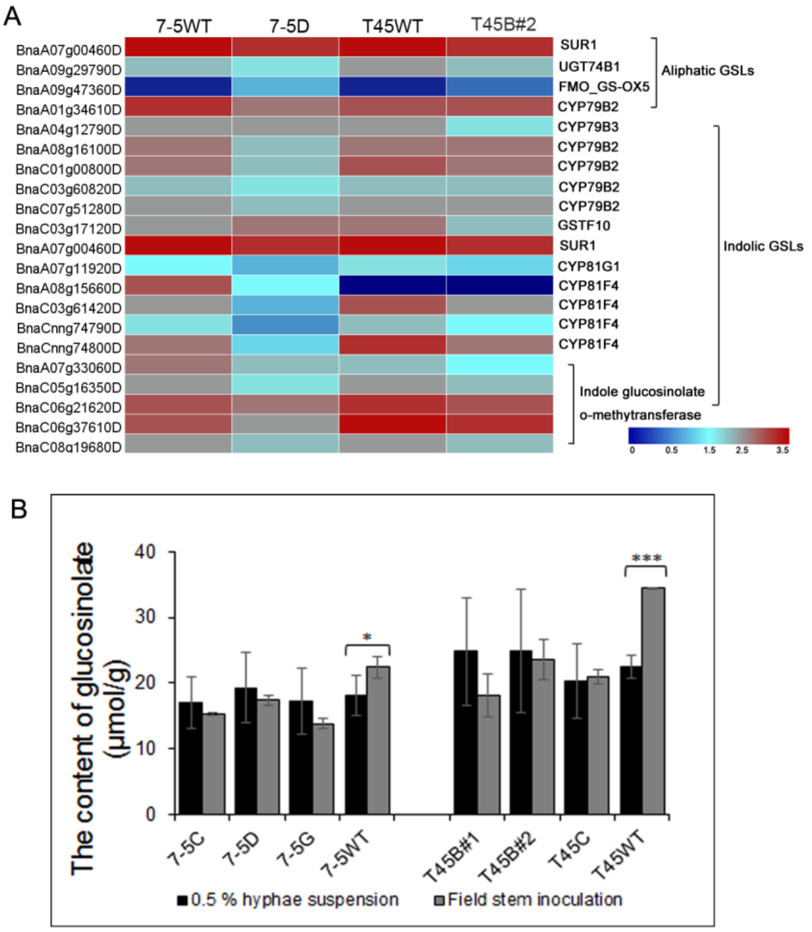

Fig. S9. The expression of GSLs biosynthesis genes and the content of seed GSLs. (A) Heatmap of the differentially expressed genes in GSLs biosynthesis. The details of the differentially expressed genes in GSLs biosynthesis pathway in T45WT, T45B#2, 7-5WT and 7-5D lines response to *S. sclerotiorum* were show in the File S3. (B) The seed GSLs content of transgenic *OsPGIP2* rapeseed evaluated after inoculation with *S. sclerotiorum*. The black columns represented the seed GSLs content of T_3 generations after infection with 0.5 % *S. sclerotiorum* hyphae suspension in the field. The grey columns showed the seed GSLs content of T_4 generations for field stem inoculation of *S. sclerotiorum*. The field assays in T_3 and T_4 generations were respectively designed in two completely randomized blocks and each replicate contained about 20 plants. WT indicates non-transformed rapeseed plants. Values are the means over nine replicates ± standard deviation. Seed GSLs content were measured based on dry matter weight. ***P < 0.001 and *P < 0.05 were tested by one-way ANOVA.

Table S1. Field testing data of individual rapeseed lines in three consecutive years.

Veriety	The length of the lesions on the stem(cm)		
	T_2	T_3	T_4
7-5C	3.12±0.24***	4.81±0.23*	3.98±0.21**
7-5D	3.89±0.24***	4.40±0.35*	4.15±0.20*
7-5G	4.43±0.27***	3.90±0.22***	4.34±0.28*
7-5WT	6.65±0.24	5.57±0.32	5.44±0.21
T45B#1	4.42±0.28***	4.40±0.29***	3.64±0.04***
T45B#2	6.43±0.21***	3.59±0.25***	4.90±0.05**
T45C	6.49±0.27***	4.70±0.33**	4.68±0.05**
T45WT	8.27±0.23	5.93±0.30	6.68±0.07

All plants were investigated the lesions length with stem inoculation at 7dpi. The length of the lesions on the stem was showed with mean values (n>30) ± standard error. The transgenic *OsPGIP2* lines were compared with their non-transformed lines, and differences within each line were tested for significance by one-way ANOVA. (*P < 0.05; **P < 0.01; ***P < 0.001). 7-5WT and T45WT are the non-transgenic controls.

Table S2. Number of clean sequence reads that map to the *Sclerotinia sclerotiorum* 1980 genome.

	Clean reads	Total mapped	Mapped rate
7-5D	47130844	4633307	9.83%
7-5WT	54035950	6965718	12.89%
T45B#2	43614190	8707432	19.96%
T45WT	39654044	10768317	27.16%

Table S3. The affinity between OsPGIP2 and *S. sclerotiorum* PGs.

	Delt G [a] (kcal/mol)	Kd [b] (mol/L)
OsPGIP2-SsPG1	-16.09	15.90e-13
OsPGIP2-SsPG3	-17.04	3.20e-13
OsPGIP2-SsPG5	-17.02	3.31e-13
OsPGIP2-SsPG6	-17.84	0.82e-13

[a] Delt G is binding free energy.

[b] Kd is dissociation constant.

The affinities about OsPGIP2 and SsPGs were predicated in PPA-Pred2 (Protein-Protein Affinity Predictor) (https://www.iitm.ac.in/bioinfo/PPA_Pred/prediction.html). The class of the protein-protein complex is set as "Enzyme-Inhibitor". The smaller the dissociation constant, the more tightly bound the ligand is, or the higher the affinity between ligand and protein.

Table S4. Summary of primers used in this study.

Primer name	Sequence（5'-3')	Purpose
333-OsPGIP2-L	CACAACAACCTGTCCGGGAG	Detection of *OsPGIP2* in transgenic families
333-OsPGIP2-R	GTAGCAGTAGGCGTCGAACC	
309-HYG	GGCGACCTCGTATTGGGAAT	Detection of *HYG* in transgenic families
309-HYG	ACCGCAAGGAATCGGTCAAT	
BnActin7-L	GGAAGCTCCTGGAATCCATGAGA	qPCR control for *B．napus*
BnActin7-R	TCTTTGCTCATACGGTCAGCAATTCC	
UBC21-L	CCTCTGCAGCCTCCTCAAGT	qPCR control for *B．napus*
UBC21-R	CATATCTCCCCTGTCTTGAAATGC	
169-OsPGIP2	TTCGCGGAGGAGACGTACTA	qPCR analysis of *OsPGIP2* expression
169-OsPGIP2	TGTGTTGGTAGCAGTAGGCG	
321-OsPGIP2-L	CAATCTCTCCGCCATCAACCT	Probe primers for southern blot
321-OsPGIP2-R	TGGCTCACGTCCACGTAGTA	
PDF1.3-L	TCATGGCTAAGGCTGCTACC	qPCR analysis of *BnaC06g22120D* expression in 7-5WT and 7-5D
PDF1.3-R	ACTCCTGACCATGTCCCACT	
CAT1-L	TACAGACACGAAACAGCA	qPCR analysis of *BnaA07g11370D* expression in 7-5WT and 7-5D
CAT1-R	GACAGAAACTAGCAAGCC	
LRR-RLKs-7L	GATTGAGGCGGAGTTTGG	qPCR analysis of *BnaA09g57210D* expression in 7-5WT and 7-5D
LRR-RLKs-7R	CTCGCAGTGCATAGGATA	
BAM1-7L	AAAACAGCGACAGAGGGT	qPCR analysis of *BnaA06g24650D* expression in 7-5WT and 7-5D
BAM1-7R	CGTGAAAGCCGAGTAAAA	
ACA4-7L	TAGTGAAAGTGGCTAGATGG	qPCR analysis of *BnaC04g47760D* expression in 7-5WT and 7-5D
ACA4-7R	CAGTGAGTGGAGCAGACC	
PRXR1-7L	GCAGTCCAGTATGTGCGTAA	qPCR analysis of *BnaA01g11800D* expression in 7-5WT and 7-5D
PRXR1-7R	CACCCTTGGAACCAGTCA	
PER21-7L	TTGCCCAAGTCCAAACCC	

PER21-7R	GACCAGGAGCCCTCTATG	qPCR analysis of *BnaC03g20530D* expression in 7-5WT and 7-5D
PER45-7L	GTGATGGCTGAGACAAAA	qPCR analysis of *BnaAnng10890D* expression in 7-5WT and 7-5D
PER45-7R	TTCAACAAGGAAAGGGTC	
EBP-7L	AGCCAAACTCAACTTCCC	qPCR analysis of *BnaC01g35070D* expression in 7-5WT and 7-5D
EBP-7R	CTCCCCACTCCACTGTAC	
EBP-7L	AGCCAAACTCAACTTCCC	qPCR analysis of *BnaA03g34290*D expression in 7-5WT and 7-5D
EBP-7R	TCCAAAACCGCAACTCAT	
AUX1-7L	TCGGTGGATGGGCTAGTGTA	qPCR analysis of *BnaC04g07210D* expression in 7-5WT and 7-5D
AUX1-7R	CAAAGGCGGTGGTGTAAAGC	
IAA7-7L	GAATCTGGCAAAATCGGCGG	qPCR analysis of *BnaC05g29300D* expression in 7-5WT and 7-5D
IAA7-7R	GCTGAGGCGACGTTGTTAAG	
LRR-7L	AGGGACCAATCCCGAGATCA	qPCR analysis of *BnaA05g20090D* expression in 7-5WT and 7-5D
LRR-7R	CAAGCCCTCAAAGGTTTGCC	
BGL2-7L	TGTTCAAACCGACCCCTGTA	qPCR analysis of *BnaC08g28170D* expression in 7-5WT and 7-5D
BGL2-7R	CCACGATTTCCAACGACC	
TUB6-7L	AATGGATACCGAACAACG	qPCR analysis of *BnaC09g44450D* expression in 7-5WT and 7-5D
TUB6-7R	TGAACTGCTCACTCACCC	
TUB1-7L	GAGAACGCTGATGAATGC	qPCR analysis of *BnaC06g36460D* expression in 7-5WT and 7-5D
TUB1-7R	ATGGGATAAGGTTGACTGC	
BGAL1-7L	AGATGACCACGGTGAAGT	qPCR analysis of *BnaA05g25660D* expression in 7-5WT and 7-5D
BGAL1-7R	AGGTGCTACGGTTACAGA	
WAT1-7L	CCGTCAAGTCGTCCATCACA	qPCR analysis of *BnaC06g22370D* expression in 7-5WT and 7-5D
WAT1-7R	AACGGCACACCATTGGGTAA	
PT525-7L	CTATGAATGTTGCTGGTG	qPCR analysis of *BnaA03g03250D* expression in 7-5WT and 7-5D
PT525-7R	CTTCTTCTGAGCCTCTTT	
NLTP2-7L	TGTCACTGGAAACTACCC	

NLTP2-7R	GAGCCTTGAGTTTTGATG	qPCR analysis of *BnaA02g09030D* expression in 7-5WT and 7-5D
MKK5-7L	CGGTTACGCTGGAGATGTGT	qPCR analysis of *BnaA01g25260D* expression in 7-5WT and 7-5D
MKK5-7R	TTCAGGCGGCTGAGACATAC	
PER54-T45-L	TTCTCCCACCATCCCTAT	qPCR analysis of *BnaA10g24230D* expression in T45WT and T45B#2
PER54-T45-R	TCAGCAGAGCCAGACCTT	
JAZ1-T45-L	TACGGCGGGCAAGTGATT	qPCR analysis of *BnaA06g13250D* expression in T45WT and T45B#2
JAZ1-T45-R	TTGGTTCGGGGTAGGAGC	
PGIP1-T45-L	GTCATTTGGGTCGTTTCC	qPCR analysis of *BnaA10g24090D* expression in T45WT and T45B#2
PGIP1-T45-R	ACGTCGTTTTGTTGGCTC	
PR4-T45-L	ACCACGGCTGACTACTGT	qPCR analysis of *BnaC03g33900D* expression in T45WT and T45B#2
PR4-T45-R	ATAGGCACTCACGGCTCT	
BGL2-T45-L	TCTCCTCTGCTCGTGAAT	qPCR analysis of *BnaC04g24490D* expression in T45WT and T45B#2
BGL2-T45-R	AGGTTTTGGTAATGGTGC	
BGAL1-T45-L	GCTGTCACGCTCATCACT	qPCR analysis of *BnaC05g39570D* expression in T45WT and T45B#2
BGAL1-T45-R	CCACGGCTAGTTTCTTCA	
BGAL4-T45-L	GTCACGCTGAAGGGAGTA	qPCR analysis of *BnaAnng30540D* expression in T45WT and T45B#2
BGAL4-T45-R	CTGGTGCGGCAAAAGTAG	
PT525-T45-L	CGGGAAGTGACGCTATGC	qPCR analysis of *BnaC09g10200D* expression in T45WT and T45B#2
PT525-T45-R	TGTGAACGGAAGGCTGAG	
NLTP4-T45-L	ACACCGTCAAGTGAAATG	qPCR analysis of *BnaCnng42990D* expression in T45WT and T45B#2
NLTP4-T45-R	CAAACTAGGACATGCTGA	
NLTP2-T45-L	CCCAACGCTCGTAAAGTC	qPCR analysis of *BnaC03g40460D* expression in T45WT and T45B#2
NLTP2-T45-R	CGCATACCAAAAGCAGGA	
UFAA1-T45-L	GACAGACAAAAGCCAATC	qPCR analysis of *BnaC07g07970D* expression in T45WT and T45B#2
UFAA1-T45-R	AGTTTCCAAGGGTAAGAG	

C-kpnI-OsPGIP2-L	TACGCGTCCCGGGGC GGTACC ATGGATGTGAAGCTCCTGC	Subclone *OsPGIP2* into JW772-CLUC
C-SalI-OsPGIP2-R	ACGAAAGCTCTGCAG GTCGAC TTATCGACGACGGCAGGCGG	
N-kpnI-SsPG1-L	ACGGGGGACGAGCTC GGTACC ATGGTTGAGATTCTTTCCTCGG	Subclone *SsPG1* into JW772-NLUC
N-SalI-SsPG1-R	CGCGTACGAGATCTG GTCGAC ACACTTGACACCAGATGGG	
N-kpnI-SsPG3-L	ACGGGGGACGAGCTC GGTACC ATGAAAATCAACAACCAACTC	Subclone *SsPG3* into JW772-NLUC
N-SalI-SsPG3-R	CGCGTACGAGATCTG GTCGAC TGCAGGGCATCCAGAAGATG	
N-kpnI-SsPG5-L	ACGGGGGACGAGCTC GGTACC ATGGTTAACCTTTCTGCCCCT	Subclone *SsPG5* into JW772-NLUC
N-SalI-SsPG5-R	CGCGTACGAGATCTG GTCGAC CAAGGAGCAAGAGACGCCA	
N-kpnI-SsPG6-L	ACGGGGGACGAGCTC GGTACC ATGCATAGAGACTTTTCCATC	Subclone *SsPG6* into JW772-NLUC
N-SalI-SsPG6-R	CGCGTACGAGATCTG GTCGAC CTTAGGACAGCCGGTTGA	
SsPG1-L	GTGGTCACGGTCTCTCCATC	qPCR analysis of *SsPG1* expression in T45WT and T45B#2
SsPG1-R	TGTCCTTGTAGGTAACGCCG	
SsPG3-L	GGAGATGCCCCTAACTCAGC	qPCR analysis of *SsPG3* expression in T45WT and T45B#2
SsPG3-R	TAGAACCAGATGTGACGGCG	
SsPG5-L	GGTCTTTCCGTTGGATCCGT	qPCR analysis of *SsPG5* expression in T45WT and T45B#2
SsPG5-R	AGGGTGATGCCTGAGTAGGT	
SsPG6-L	TGTTACGAGCGGGAACAACA	qPCR analysis of *SsPG6* expression in T45WT and T45B#2
SsPG6-R	GGTGGTTCCTTCGTTGGACT	

SsH3-L	ATGGCTCGTACCAAGCAAAC	qPCR control for *S. sclerotiorum*
SsH3-R	AGAGCACCAATAGCGGAAGA	
28rDNA-L	CTGAAAGGCCTGTGAGCACT	qPCR control for *S. sclerotiorum*
28rDNA-R	CCCATTGCCGTCTAGTCTGT	

The underscore indicates the cleavage site.
